# Discovering miRNA Regulatory Networks in Holt–Oram Syndrome Using a Zebrafish Model

**DOI:** 10.3389/fbioe.2016.00060

**Published:** 2016-07-14

**Authors:** Romina D’Aurizio, Francesco Russo, Elena Chiavacci, Mario Baumgart, Marco Groth, Mara D’Onofrio, Ivan Arisi, Giuseppe Rainaldi, Letizia Pitto, Marco Pellegrini

**Affiliations:** ^1^Laboratory of Integrative Systems Medicine (LISM), Institute of Informatics and Telematics (IIT), Institute of Clinical Physiology (IFC), National Research Council (CNR), Pisa, Italy; ^2^Department of Computer Science, University of Pisa, Pisa, Italy; ^3^Institute of Clinical Physiology (IFC), National Research Council (CNR), Pisa, Italy; ^4^Leibniz Institute on Ageing, Fritz Lipmann Institute (FLI), Jena, Germany; ^5^Genomics Facility, Fondazione EBRI Rita Levi-Montalcini, Roma, Italy

**Keywords:** zebrafish, heart, microRNA, NGS, microarray, Holt–Oram, data integration

## Abstract

MicroRNAs (miRNAs) are small non-coding RNAs that play an important role in the post-transcriptional regulation of gene expression. miRNAs are involved in the regulation of many biological processes such as differentiation, apoptosis, and cell proliferation. miRNAs are expressed in embryonic, postnatal, and adult hearts, and they have a key role in the regulation of gene expression during cardiovascular development and disease. Aberrant expression of miRNAs is associated with abnormal cardiac cell differentiation and dysfunction. Tbx5 is a member of the T-box gene family, which acts as transcription factor involved in the vertebrate heart development. Alteration of Tbx5 level affects the expression of hundreds of genes. Haploinsufficiency and gene duplication of Tbx5 are at the basis of the cardiac abnormalities associated with Holt–Oram syndrome (HOS). Recent data indicate that miRNAs might be an important part of the regulatory circuit through which Tbx5 controls heart development. Using high-throughput technologies, we characterized genome-widely the miRNA and mRNA expression profiles in WT- and Tbx5-depleted zebrafish embryos at two crucial developmental time points, 24 and 48 h post fertilization (hpf). We found that several miRNAs, which are potential effectors of Tbx5, are differentially expressed; some of them are already known to be involved in cardiac development and functions, such as miR-30, miR-34, miR-190, and miR-21. We performed an integrated analysis of miRNA expression data with gene expression profiles to refine computational target prediction approaches by means of the inversely correlation of miRNA–mRNA expressions, and we highlighted targets, which have roles in cardiac contractility, cardiomyocyte proliferation/apoptosis, and morphogenesis, crucial functions regulated by Tbx5. This approach allowed to discover complex regulatory circuits involving novel miRNAs and protein coding genes not considered before in the HOS such as miR-34a and miR-30 and their targets.

## 1 Introduction

MicroRNAs (miRNAs) are small non-coding RNAs of about 20–23 nucleotides that play an essential role in a variety of biological important pathways from development and physiology to diseases such as cancer (Chen and Rajewsky, 2007; Small and Olson, 2011). miRNAs are mostly known to function by targeting complementary sequences in mRNA transcripts, usually in the 3′ untranslated region (3′ UTR) and so inhibiting the translation and altering the stability of mRNA (Bartel, 2004; Yates et al., 2013). The identification and validation of miRNA–mRNA interactions is fundamental for discerning the role of miRNAs in the complex context of regulatory networks. However, since the miRNA binding is mostly not a perfect one-to-one match with the complementary target sites, it is difficult to predict miRNA targets. Consequently, several computational methods and tools have been developed in the last years (Yue et al., 2009; Peterson et al., 2014). They encompass a range of different computational approaches, from the modeling of physical interactions, exploiting common features like seed match, conservation, free energy, and site accessibility to the incorporation of less common features extracted through machine learning techniques. Computational methods predict hundreds of thousands target mRNAs per miRNA, instead the number of experimentally validated targets is very low. One possibility to reduce the false positive rate is to combine high-throughput experimental data with sequence-based predictions (Huang et al., 2007; Muniategui et al., 2013). Although, this approach does not allow to identify miRNA targets that are repressed exclusively at the translational level. Since many miRNAs cause degradation of their targets (Baek et al., 2008; Hendrickson et al., 2009; Guo et al., 2010; Subtelny et al., 2014), the integration of expression profiles has been proposed to be an effective strategy to discover true miRNA–target interactions (Gennarino et al., 2009; Nazarov et al., 2013; Albert et al., 2014).

In this work, we used expression values of miRNAs and mRNAs obtained with high-throughput technologies to study complex regulatory networks altered in the Holt–Oram syndrome (HOS). HOS is a rare autosomal congenital disease characterized by cardiac and upper limb malformations (Basson et al., 1997). Mutations in the T-box gene Tbx5, which encodes a key transcription factor for vertebrate heart development, are responsible for HOS (Horb and Thomsen, 1999; Goetz et al., 2006). Family members with identical Tbx5 mutations can display large variations in malformation severity and HOS penetrance. This peculiar characteristic of HOS can be explained by the observation that Tbx5 is part of an extremely complex regulatory network. Due to the high number of messenger RNAs that are targeted by one miRNA, miRNAs are the best candidates to orchestrate the downstream regulation of Tbx5 gene expression in embryonic heart development. We have recently shown that miRNAs are crucial components of this network (Chiavacci et al., 2012, 2015). In fact, we proved that in mouse cardiac cells and zebrafish embryos, Tbx5 is able to regulate several miRNAs and, in particular, miR-218 and miR-19 (Chiavacci et al., 2012, 2015). The dysregulation of both miRNAs has a severe impact on heart development, affecting early heart morphogenesis.

As a model system, zebrafish has been extensively used for studying early vertebrate development (Kimmel et al., 1995; Yao et al., 2014) over the last 20 years. In particular, the HOS model called heartstring (hts) mutant has been well established in zebrafish, and it recapitulates almost completely the HOS characteristics. Furthermore, the zebrafish hts mutant can be easily replicated with the injection in zebrafish eggs of a specific Tbx5 morpholino (small antisense ribo-oligonucleotides, which blocks target translation) (Garrity et al., 2002).

Here, we propose an integrative approach, which uses experimental data from zebrafish HOS model system and computational methods for investigating *in vivo* complex regulatory networks perturbed in this pathology across two different stages of zebrafish development, 24 and 48 hpf. Those two stages were chosen since they mark fundamental steps in heart development. By 24 hpf, the migration phase is concluded, and the heart tube lies along the anteroposterior axis of the embryo with the atrial end to the left of the midline. By 48 hpf, the heart development is substantially completed: the heart terminated the looping phase and functional valves are formed (Kimmel et al., 1995; Yao et al., 2014). We show that it is possible to use data integration methods for studying rare diseases, providing significant insight into biological processes, and identifying new potential markers and drug targets of clinical interest.

## 2 Materials and Methods

### 2.1 Embryos Injection

The zebrafish line used in this study is the wild-type AB strain, the animals were raised and maintained under standard laboratory conditions (Westerfield, 1993). To silence the zebrafish gene, Tbx5a we used the antisense morpholino oligonucleotide MO-Tbx5a against the translational start site of the gene, the sequence of MO-Tbx5a was 5′-GAA AGG TGT CTT CAC TGT CCG CCA T-3′ (Garrity et al., 2002). The sequence of the control morpholino, MO-Ct, was 5′-CCT CTT ACC TCA GTT ACA ATT TAT A-3′. All morpholinos were supplied by Gene Tools LLC. Zebrafish morpholinos were injected into the yolk of 1-cell stage embryos with a constant injection volume, ~1 nl, using a microinjector (Tritech Research, Los Angeles, CA, USA). Zebrafish eggs were injected with 1.5 ng of MO-Tbx5a or 1.5 ng of MO-Ct, and embryos were collected at 24 and 48 hpf.

### 2.2 RNA Extraction, Library Preparation, Sequencing, and Microarray

For high-throughput DNA sequencing, total RNA was extracted from batch of *n* = 50 zebrafish embryos. The library preparation was done as described in (Baumgart et al., 2012). In detail, 500 ng of total RNA was used as input material. Library preparation was done using the TruSeq Small RNA Sample Prep (Illumina). The purified libraries were quantified on the Agilent DNA 1000 chip, diluted to 10 nM and subjected to sequencing-by-synthesis on Illumina HiSeq 2000 producing single-end 51 bp read length. Two independent batches of embryos were used for MO-Tbx5a and MO-Ct at 24 hpf, one for both condition at 48 hpf.

To measure mRNA expression, the Agilent Low Input Quick Amp labeling kit was used to retrotranscribe into the cDNA (from 200 ng total RNA), amplify, and incorporate the cyanine 3-labeled CTP (cRNA). The method uses the T7 RNA polymerase, which simultaneously amplifies and incorporates cyanine 3-labeled CTP. The fluorescent cRNA was purified and hybridized to the Agilent Zebrafish V3 Gene Expression Microarray 4 × 44, according to the manufacturer protocol. Three independent batches of embryos were assessed for MO-Tbx5a, while two for MO-Ct both at 24 and 48 hpf stages. Resulting images were quantified and text files containing raw values were analyzed.

### 2.3 Analysis of Sequencing and Microarray Data

Raw sequences were obtained and de-multiplexed using the Illumina pipeline CASAVA v1.8.2 FastQC v0.10.1^1^, which was used for quality check, and primary reads were initially trimmed off to remove adapters sequence using Cutadapt v.1.2.1 (Martin, 2011). Employing FASTX_Toolkit (0.0.13.1), the reads with N calls were discarded. Remaining high quality reads, with a minimum length of 17 bp and a maximum 38 bp after clipping, were clustered for unique hits and mapped to zebrafish pre-miRNA sequences present into the mirBase (release 20) employing miRExpress (v2.1.3; Wang et al., 2009). We allowed 95% of sequence identity between read and reference sequence and a length tolerance range of 4 bp for mapping. miRNAs expression profiles were built by calculating the sum of read counts for each miRNA according to the alignment criteria. Differential expression analysis of miRNAs identified by miRExpress was performed using Bioconductor’s package DESeq (Anders and Huber, 2010). The reads count, used as measure of miRNAs quantification, was first normalized by library size factors to a common scale. The analysis was then performed and *p*-values were estimated using a negative binomial distribution model and local regression to estimate the relationship between the dispersion and the mean of each miRNAs. Raw *p*-values were finally adjusted for multiple testing using the Benjamini and Hochberg (1995) procedure controlling the false discovery rate (FDR). miRNAs with an adjusted *p*-value <0.05 were considered to be differentially expressed.

For microarrays, pre-processing of the data included background correction using a normal-exponential convolution model (offset = 16) (Ritchie et al., 2007) and cyclic loess normalization (Ballman et al., 2004) implemented in Limma package v.3.14.4 (Smyth, 2004). Low-expressed probes were filtered out keeping probes that are at least 10% brighter than the 95th percentile of the negative controls on at least 2 arrays. The Agilent Single channel Expression Microarray 4 × 44K for Zebrafish contains 39344 probes, 39162 are unique. For 35073 of them, we were able to retrieve gene accession ids corresponding to 21956 unique gene IDs. The linear modeling approach and empirical Bayes statistics implemented by Limma were used for assessing differentially expression. Finally, *p*-values were adjusted for multiple testing by means of the Benjamini and Hochberg method to control the false discovery rate. Genes with FDR less than 0.05 and fold change (FC) higher than 1.3 were selected for downstream analysis.

All statistical analyses were conducted using *R* and available Bioconductor packages.^2^

### 2.4 Integrated Analyses of Zebrafish miRNA and mRNA Expression Profiles

**Figure 1:**
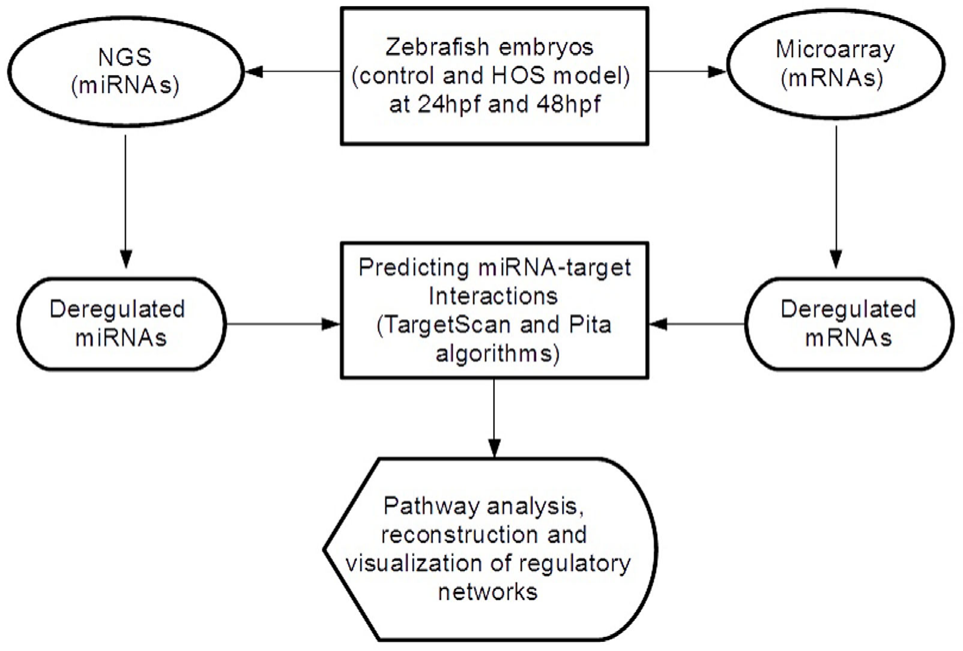
**Overview of the analytical workflow used in the study to identify inversely correlated putative target genes and to build altered regulatory networks in HOS**.

In order to discover miRNA-target pairs involved in HOS, we combined inverse correlations between miRNA and mRNA expression for improving *in silico* microRNA target predictions (see Figure 1). We selected the significant differential expressed miRNAs and mRNAs and performed target prediction analysis. Since miRNAs act at the post-transcriptional level downregulating their targets binding on the 3′-UTR of mRNAs, in this study, we focused our attention on these sequences that we retrieved from the UCSC Table Browser.^3^ We predicted miRNA target sites in the 3′-UTR using TargetScan Fish 6.2 (Lewis et al., 2005) and Pita (Kertesz et al., 2007) algorithms and then selected the consensus. Finally, we extracted the inversely correlated interactions (to reflect the typical miRNA–mRNA relationship) obtaining the final miRNA-target list.

## 3 Results

In the following sections, we detail the expression profiles of both miRNAs and annotated genes, which resulted altered by Tbx5a depletion during early zebrafish developmental stages (24 and 48 hpf). Small RNAseq and microarray analysis were performed to generate, respectively, miRNA and mRNA profiles. Moreover, we describe the main results obtained by integrating experimental data with computational methods to investigate *in vivo* regulatory networks modified by Tbx5 dosage alteration.

### 3.1 Sequencing and Annotation of miRNAs Modulated by Tbx5a at 24 and 48 hpf

**Figure 2:**
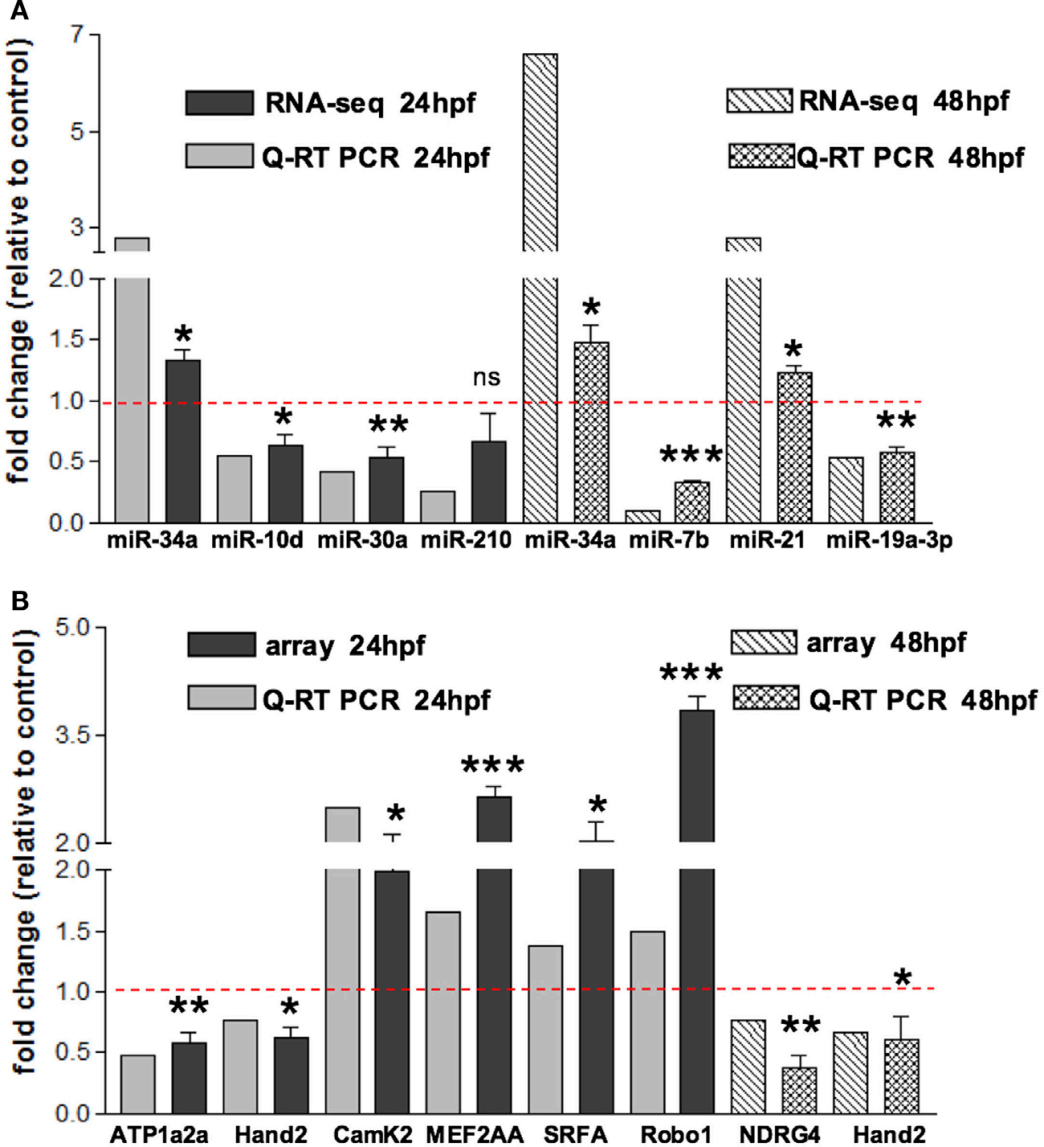
**Validation of small RNA seq profiling and array-based gene expression profiles by Quantitative RT-PCR**. **(A)** Sequencing and corresponding Q-RT PCR expressions of eight of the miRNAs reported in Table 1 and identified as differentially modulated in MO-Tbx5 vs. MO-Ct embryos at 24 hpf and at 48 hpf are reported. Values are expressed as fold change of MO-Tbx5 relative to MO-Ct. For Q-RT PCR, values are normalized on U6 expression. As pointed out in the results, miR-19a RNA-seq value is from Chiavacci et al. (2015). miR-210 is the 5p isoform. **(B)** Microarray and corresponding qRT-PCR expressions of eight genes showing differential expression in MO-Tbx5 compared to MO-Ct embryos at 24 and 48 hpf are reported. Values are expressed as fold change of MO-Tbx5 relative to MO-Ct. For Q-RT PCR, values are normalized on EF1, beta actin, and 18S expression. The values reported in the Q-RT PCR analysis are the mean of at least three independent microinjection experiments, *t*-test was used for statistical analysis: *p < 0.05, **p < 0.01, and ***p < 0.001.

In order to assess miRNAs expression modulation in zebrafish embryos after Tbx5a depletion, we conducted massive parallel sequencing experiments producing between 12.7 and 25.1 million total sequencing reads were obtained for each given library (16.8 mean) and this ranged from 10.5 to 20.2 million reads of 17–38 length after adapter trimming. On average, around 760 thousands of reads mapped to zebrafish miRNAs, annotated in miRBase v20 identifying 367 mature miRNAs on average per sample (see Table S2 in Supplementary Material). Among them, 19 miRNAs resulted to be significantly modulated at 24 hpf and 33 at 48 hpf (Table S3 and S4 in Supplementary Material respectively). We selected the most variable miRNAs, in terms of expression fold-change between Tbx5 and Ct morphants for downstream analysis: miR-34a, miR-10d-5p, miR-30a, miR-210-3p, and miR-5p at 24 hpf, miR-34a, miR-462, miR-146a, miR-21, miR-7b, and miR-190b at 48 hpf (Table 1). Differently from experiments reported in our previous work (Chiavacci et al., 2015), the downregulation of miR-19a at 48 hpf was not significant. However, this miRNA was included in the list of miRNAs modulated by Tbx5 because: (1) Q-RT PCR analysis performed in four different sets of experiments confirmed miR-19a downregulation (Figure 2B), (2) this downregulation was clearly supported by physiological data and by *in situ* hybridization experiments already presented (Chiavacci et al., 2015). Besides miR-19a-3p, other seven differentially modulated miRNAs were measured by quantitative RT-PCR and fold-changes were compared in Figures 2A,B for 24 and 48 hpf, respectively. All modulation was confirmed except for miR-210-5p, which resulted not significative.

**Table 1:** **Selected differentially expressed miRNAs at 24 and 48 hpf**.

Devel. stage	miRNA	FC	p-val	adj p-val
24 hpf	dre-miR-34a	2.82	1.03e−12	2.99e−10
	dre-miR-10d-5p	0.55	11.24e−07	6.77e−06
	dre-miR-30a	0.41	9.40e−12	1.02e−09
	dre-miR-210-3p	0.33	8.29e−12	1.02e−09
	dre-miR-210-5p	0.26	1.68e−10	1.22e−08
48 hpf	dre-miR-34a	6.62	7.43e−16	2.70e−14
	dre-miR-462	5.6	5.95e−10	7.63e−09
	dre-miR-146a	4.51	1.05e−09	1e27e−08
	dre-miR-21	2.84	1.65e−10	2.25e−09
	dre-miR-19a-3p^a^	0.68	8.52e−02	4.91e−02
	dre-miR-7b	0.10	1.83e−07	1.66e−06
	dre-miR-190b	0.01	1.21e−18	5.29e−17

*^a^Data for miR-19a-3p comes from our previous published data in (Chiavacci et al., 2015)*.

### 3.2 Tbx5 Sensitive Genes in Early Developmental Stages of Zebrafish

To characterize the gene expression profiles at 24 and 48 hpf of zebrafish development and evaluate how altered Tbx5 dosage influences the genome-wide transcription, we measured mRNAs using expression microarray technology (see Materials and Methods for details). mRNAs were extracted from zebrafish embryos injected with MO-Tbx5a or MO-Ct and collected at 24 and 48 hpf. Using an absolute FC cut-off of 1.3 and an adjusted *p*-value of 0.05, we identified 7100 differentially modulated genes after TBx5a silencing at 24hpf, while 2276 genes at 48 hpf. The magnitude of differential expression was formally tested to be biologically significant using the *t*-test relative to a threshold (TREAT) method (McCarthy and Smyth, 2009) implemented in Limma. The complete lists are available as Supplementary Material. Validation by relative Q-RT PCR was performed for some of the genes taking into consideration the relevance for the cardiac context. Q-RT PCR analysis confirmed the microarray data (Figure 2A for 24 hpf and Figure 2B for 48 hpf).

To highlight most relevant functional categories among identified modulated genes, we performed a Functional Annotation Clustering using The Database for Annotation, Visualization and Integrated Discovery (DAVID) tool (Da Wei Huang and Lempicki, 2008). The Functional Annotation Clustering integrates the Kappa statistics to measure common genes between two annotations (e.g., ontological terms), and the fuzzy heuristic clustering to classify the groups of similar annotations according to kappa values. The resulting groups have similar biological meaning due to share similar gene members. We considered KEGG pathways and Gene Ontology terms performing the enrichment analysis of downregulated and upregulated genes (separately) at 24 and 48 hpf. Clusters that resulted significant (*p* < 0.05) are reported in Table 2. Interestingly, at 24 hpf, the top scoring functional categories contained genes that are involved in cell adhesion and ion binding. It is well known that morphogenesis requires specific cell adhesion molecules that are expressed in a precise developmental time, and the altered gene expression leads to heart defects (Buck et al., 1993; Kwee et al., 1995). In accordance with this observation and with our results, genes annotated with the term homophilic cell adhesion were also identified as significantly upregulated following inhibition of Tbx5a in a microarray-based expression profile performed in 56 hpf Tbx5 morphant zebrafish embryos (Mosimann et al., 2015). Furthermore, we found that the majority of modulated genes consisted of the cation and ion binding categories. In this context, the cation calcium has an important role in heart development, functions, and diseases (Arnolds et al., 2012; Crocini et al., 2014). The Calcium Binding Proteins (CaBPs) share a very similar domain organization with Calmodulin (CaM) and have been shown to have coevolved in vertebrate animals (McCue et al., 2010). The CaBPs have an important role during the development and in several diseases, such as Diastolic dysfunction that is characterized by slow or incomplete relaxation of the ventricles during diastole and is an important player in heart failure pathophysiology (Asp et al., 2013).

**Table 2:** **Most significant categories from functional annotation clustering analysis of the deregulated transcripts were reported**.

Time-regulation	Cluster	Term	Benjamini *p* value	Fold-enrich.
24 hpf, up genes	c1	GO:0007155 cell adhesion	6.97e−03	2.11
	c1	GO:0022610 biological adhesion	6.97e−03	2.11
	c1	GO:0007156 homophilic cell adhesion	8.87e−03	3.27
	c1	GO:0016337 cell–cell adhesion	1.28e−02	2.89
	c2	GO:0008270 zinc ion binding	5.71e−03	1.33
	c2	GO:0046914 transition metal ion binding	4.12e−03	1.28
	c3	GO:0006468 protein amino acid phosphorylation	1.24e−02	1.67
	c3	GO:0016310 phosphorylation	2.51e−02	1.57
24 hpf, down genes	c1	GO:0044429 mitochondrial part	2.56e−03	1.81
	c1	GO:0005739 mitochondrion	3.97e−03	1.62
	c1	GO:0031975 envelope	2.07e−02	1.59
	c1	GO:0031967 organelle envelope	2.10e−02	1.60
	c1	GO:0019866 organelle inner membrane	2.33e−02	1.88
	c1	GO:0005743 mitochondrial inner membrane	2.40e−02	1.88
	c1	GO:0005740 mitochondrial envelope	2.55e−02	1.74
	c1	GO:0031966 mitochondrial membrane	2.97e−02	1.71
	c2	GO:0046872 metal ion binding	4.10e−02	1.19
	c3	GO:0004672 protein kinase activity	4.90e−02	1.56
48 hpf, down genes	c1	GO:0043565 sequence-specific DNA binding	1.02e−08	2.25
	c1	GO:0003700 transcription factor activity	1.24e−06	1.94
	c1	GO:0030528 transcription regulator activity	2.57e−06	1.76
	c1	GO:0051252 regulation of RNA metabolic process	7.00e−05	1.71
	c1	GO:0006355 regulation of transcription. DNA-dependent	1.07e−04	1.72
	c1	GO:0003677 DNA binding	3.12e−03	1.44
	c1	GO:0045449 regulation of transcription	7.05e−03	1.46
	c2	GO:0019825 oxygen binding	1.07e−04	9.26
	c2	GO:0005344 oxygen transporter activity	1.07e−04	9.26
	c2	GO:0005833 hemoglobin complex	2.57e−04	10.68
	c2	GO:0015669 gas transport	4.79e−04	8.92
	c2	GO:0015671 oxygen transport	4.79e−04	8.92
	c3	dre00010: glycolysis/gluconeogenesis	2.85e−03	3.99

### 3.3 Changes in miRNA Expression and Integration with mRNA Profile Identify Potential miRNA–mRNA Target Pairs Involved in HOS

**Figure 3:**
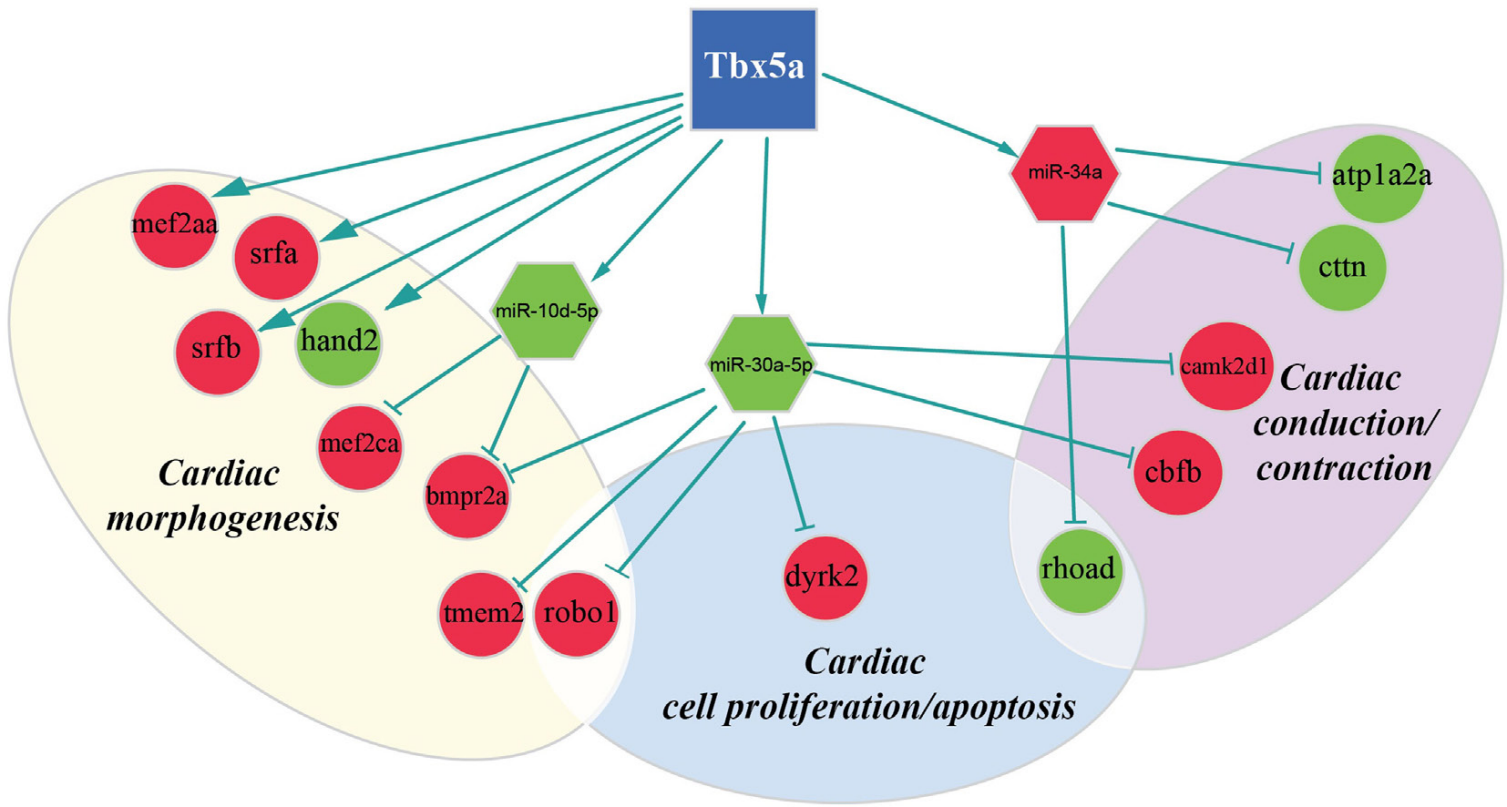
**Regulatory network altered in zebrafish HOS model at 24 hpf**. Potential interactions involving Tbx5, transcriptional factors, miRNAs, and their targets are shown together with the functional impact in heart development.

**Figure 4:**
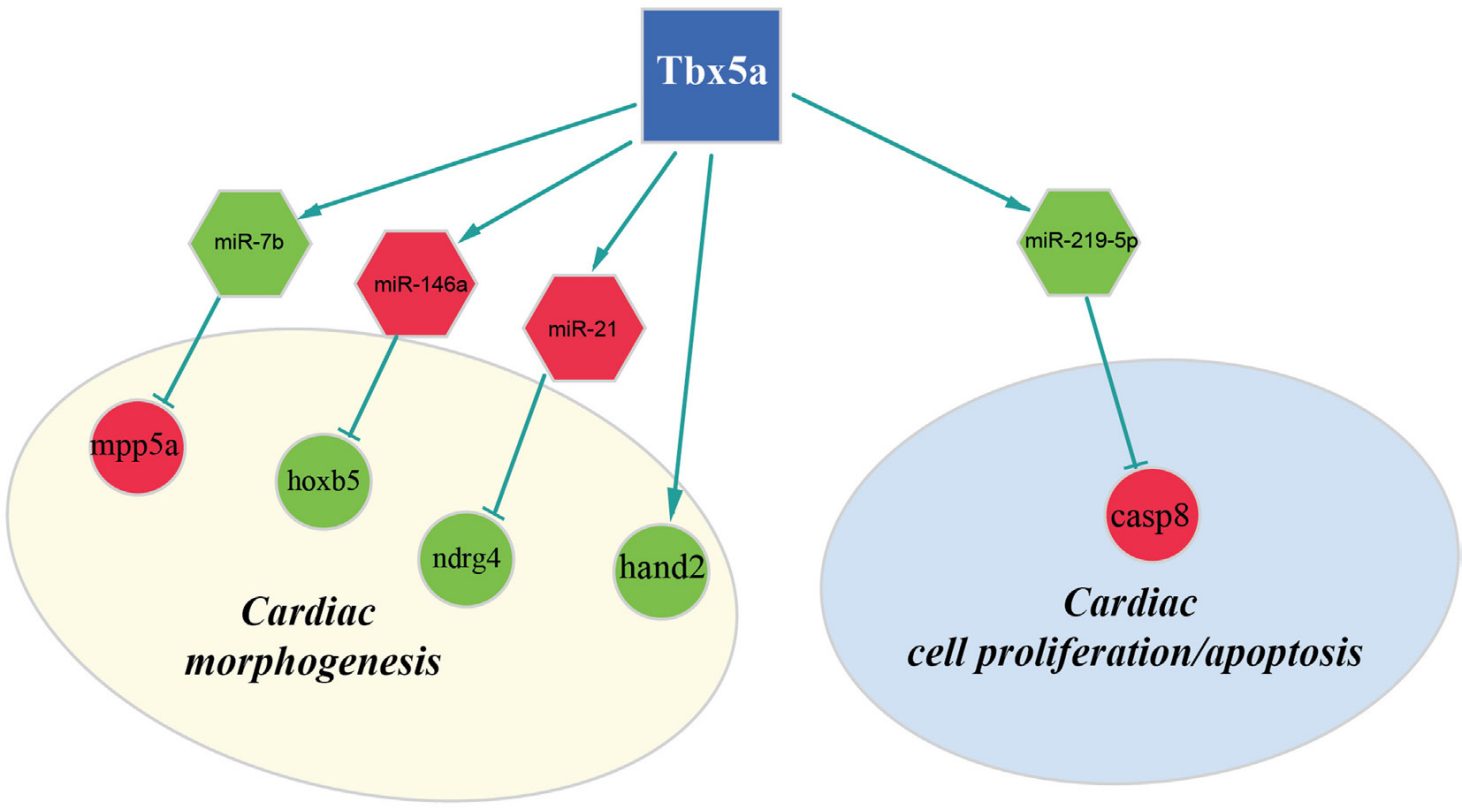
**Regulatory network altered in zebrafish HOS model at 48 hpf**. Potential interactions involving Tbx5, miRNAs, and their targets are shown together with the functional impact in heart development.

In this study, we integrated two target prediction algorithms, TargetScan and Pita, with miRNA and gene expression data to refine *in silico* predictions and reduce the number of false positive interactions. The resulted miRNA-target pairs consisted of 122 potential targets at 24 hpf for upregulated miRNAs and 372 potential targets for downregulated miRNAs (complete lists are in Table S5 and S6 in Supplementary Material). At 48 hpf, we discovered 142 potential targets for upregulated miRNAs and 162 for downregulated miRNAs (see Table S7 and S8 in Supplementary Material). Among them, several miRNA–mRNA interactions involved genes that are known to be connected to heart development or cardiac functions published in previous works (Table S9 and S10 in Supplementary Material). We summarized these finding in Figures 3 and 4 and explore most interesting functional relations in the next section.

## 4 Discussion

Tbx5 is a crucial transcription factor in heart development. In HOS murine model, it has been shown that even small alterations of this gene cause modulation of hundreds of genes (Mori et al., 2006). It has been suggested that the strong impact that Tbx5 has on gene expression is mainly the result of its ability to modulate other regulators, such as different transcription factors, in a very complex regulatory network. Our previous studies suggest that Tbx5 affects the embryo development by modulating also miRNAs (Chiavacci et al., 2012). Moreover, the fact that miR-19a replacement is able to partially rescue fins and cardiac defects in a zebrafish model of HOS, strongly supports the importance of miRNAs in Tbx5 regulatory circuits (Chiavacci et al., 2015).

In this study, we analyzed miRNA and mRNA expression profiles at two fundamental time points (24 and 48 hpf) of zebrafish embryos development after depletion of Tbx5 and compared them to the wild-type ones. We employed expression data to improve miRNA-target predictions of computational sequence-based methods by means of anticorrelation analysis of miRNA–mRNA expression levels. Repression by animal miRNAs, differently from plant miRNAs, leads to decreased translational efficiency and/or decreased mRNA levels. Although, the relative contributions of these two outcomes is still unknown and increasing experimental evidences show that changes in mRNA levels closely reflects the impact of miRNAs on gene expression suggesting that destabilization of target mRNAs by exonucleolytic activity is the main mechanism to decrease protein output (Baek et al., 2008; Hendrickson et al., 2009; Guo et al., 2010; Subtelny et al., 2014). Therefore, the anticorrelation analysis of miRNA–mRNA expression levels may contribute to elucidate large portion of miRNA–mRNA regulatory networks affected by pathological conditions. This approach allowed us to identify putative miRNA–target interactions, and cardiac transcription factors that are particularly interesting in the context of HOS.

One of the most interesting miRNA identified as upregulated both at 24 and 48 hpf was miR-34a. MiR-34 family members (miR-34a, -34b, and -34c) are upregulated in the heart in response to stress and the silencing of the entire miR-34 family could protect the heart against pathological cardiac remodeling and improve cardiac functions (Bernardo et al., 2012). Moreover, miR-34a is induced in the aging heart and *in vivo* silencing of miR-34a reduces age-associated cardiomyocyte cell death. The inhibition of miR-34a reduces cell death and fibrosis following acute myocardial infarction and improves recovery of myocardial function (Boon et al., 2013). These recent studies show an emerging role of miR-34a (and the miR-34 family) as potential regulator of heart remodeling. Therapies that inhibit miR-34a could be useful for cardiac pathologies and HOS.

We discovered several potential miR-34a targets with a possible connection with heart development and HOS (Figure 2). The *ATPase Na*^+^*/K*^+^ *transporting, alpha 2a polypeptide (ATP1a2a)* transcript is downregulated in 24 hpf Tbx5a morphants. Since the ATP1a2a contributes to the Ca homeostasis by pumping sodium ions (Na^+^) out of cells and potassium ions (K^+^) into cells, a downregulation of this enzyme might have a negative impact in cardiac contractility and control of arrhythmias. This observation is consistent with the crucial role of Tbx5 in the regulation of cardiac contraction in embryos and in adults. Interestingly, HOS patients show diastolic filling abnormalities and downregulation of ATP2a2, which regulates Ca fluxes in the SR (Zhu et al., 2008).

Furthermore, our data suggest that miR-34a might impact cardiac contraction by regulating a member of the *ras homolog gene family*, rhoad. RhoA, controlling the Rho-kinase pathway, plays an important role in various fundamental cellular functions, including contraction and motility (Satoh et al., 2011). Moreover, in line with the pro apoptotic role exerted by miR-34a (Raver-Shapira et al., 2007), we observed upregulation of *dual-specificity tyrosine-(Y)-phospho-regulated kinase 2 (dyrk2)*, putative miR-30a target, which negatively regulates the cardiomyocyte growth by mediating repressor function of GSK-3 beta on eIF2B (Weiss et al., 2013) and upregulation of *caspase 8*, putative target of miR-219-5p downregulated at 48 hpf (Figure 3).

As expected, several genes affecting cardiac morphogenesis were identified in our analysis (Figures 2 and 3). Specifically *Roundabout Guidance Receptor 1 (Robo1)*, which is involved in heart tube formation in zebrafish (Fish et al., 2011) and tmem2, whose expression in myocardial and endocardial tissues in zebrafish and mouse is required for regionally restrict atrioventricular canal boundary and endocardial cushion development. Both genes are putative miR-30a targets at 24 hpf.

Recently, a role for Tbx5 in the establishment of correct heart asymmetry in zebrafish embryos has been highlighted (Pi-Roig et al., 2014). Our data suggest that miR-30a and miR-10d might be contribute to this specific Tbx5 function by controlling respectively bmpr2a (Monteiro et al., 2008) and camk2d1 (Francescatto et al., 2010).

Another interesting miRNA that we found upregulated at 48 hpf is miR-21 whose deregulation in heart has been reported to contribute to cardiovascular disease (Jazbutyte and Thum, 2010). More recently, a crucial role of this miRNA in heart valve formation has been also shown in zebrafish (Banjo et al., 2013), and the alteration of cardiac valve morphology is one of the hallmark of zebrafish HOS phenotype (Camarata et al., 2010; Chiavacci et al., 2012). Two predicted targets of miR-21 are NDRG1B and NDRG4, members of the *N-myc downstream regulated gene (NDRG) family*, which are downregulated in Tbx5 morphants at 48 hpf. Alterations of NDRG4 cause several of the cardiac defects that characterize the heartstring mutants and are significantly decreased in hearts with reduced Tbx5 activities (Qu et al., 2008). Therefore, we hypothesized that Tbx5 might affect NDRG4 expression through miR-21 modulation.

Although in this study, we used whole embryos for our analysis, we discovered important alterations on transcription factors with crucial roles in heart development. In particular, we observed downregulation both at 24and 48 hpf of the *bHLH transcription factor Hand2* (Yelon et al., 2000). Mutations in the hands off locus, which encodes for this transcription factor, cause defects in myocardial development in an early stage, produce a reduced number of myocardial precursors, and show delayed differentiation of the pectoral fin mesenchyme (Schindler et al., 2014). All these phenotypic characteristics are in line with the observed Tbx5 morphant phenotype. In HOS mouse hearts, a strong downregulation of Hand1 was observed (Mori et al., 2006). In mouse, Hand1 and Hand2 are members of the *Hand subfamily* and have partially redundant functions (Yelon et al., 2000; Tamura et al., 2014). However, in zebrafish, only one member of the hand family has been identified, the Hand2 transcription factor, which is able to perform several of the functions that in mammals are Hand1 specific (Togi et al., 2006; Reichenbach et al., 2008). Therefore, modulation of Hand1 in mouse or Hand2 in zebrafish might have similar functional consequences. Indeed, it has been shown that Hand2 is able to downregulate Nppa, a direct target of Tbx5 and Irx4 an other important cardiac transcription factor strongly downregulated in HOS mouse heart (Bruneau et al., 2001; Mori et al., 2006). In our analysis, we were not able to detect a significant modulation of Nppa gene whose expression is restricted to the cardiac tissue. On the contrary, at 48 hpf, we detected a downregulation of Irx4. This gene is not only expressed in the heart tissues but also present in the eye, a district which is relatively large at this time of development and where Tbx5 is functionally active.

Differently from Hand2, MEF2AA, MEF2CA, SRFB, and SRFA resulted upregulated in 24 hpf embryos depleted for Tbx5a. Among them, MEF2CA is a putative target of miR-10d, both miRNAs already mentioned as downregulated at 24 hpf. All of them codify for transcription factors largely expressed in mesodermal tissues and involved in cardiac developmental patterns highly active at 24 hpf. Consequently, alterations in the expression of these factors have important effects on cardiac development. However, it is hard to predict whether dysregulation of these genes might have positive or negative regulatory effects on their targets. For Tbx5 direct interactors, such as MEF2CA, the ratio between interactors seems more important than the absolute level of the specific factor (Takeuchi et al., 2011). For SRFs, it has been shown that a mild increase may pose either positive and/or negative modulatory effects on their target activation depending on the cofactor recruited (Miano, 2003; Zhang et al., 2003). Interestingly, a negative functional cooperator of SRF is SRFBP1 that we identified as upregulated in 24 hpf Tbx5a morphants. SRFBP1 is highly expressed in fetal and adult mouse heart and functionally interacts with SRF and myocardin in repressing the atrial natriuretic factor promoter activity (Zhang et al., 2004). The data suggest that the observed mild increase of SRF and SRFBP1 in zebrafish Tbx5a morphants might contribute in the downregulation of Nppa, which characterizes the HOS disease.

In conclusion, in this study, we proposed an integrative analysis of miRNA and mRNA expression profiles in a zebrafish model to study the impact of the downregulation of Tbx5 responsible of the HOS. We found several deregulated transcripts including important transcription factors for heart development and diseases, and several deregulated miRNAs with a potential role in the pathology. This model uncovered novel miRNAs and protein coding genes not considered before in the HOS such as miR-34a and miR-30 and their targets. Further dissection of these regulatory circuits will shed light on fundamental pathways in heart development that can contribute to the pathogenesis of human heart diseases. Identification of new TBX5 targets might not only help understand the complexity of HOS phenotype but also contribute in finding novel therapeutic strategies to treat congenital disease. Future experiments are needed to test the role of the identified miRNAs regulated by Tbx5 and the effects on their downstream targets.

### 4.1 Data Accession Codes

The data discussed in this publication have been deposited in NCBI’s Gene Expression Omnibus and are accessible through GEO Super Series accession number GSE64466.^4^

## Author Contributions

RD and LP conceived the work and interpreted the data; RD analyzed sequencing and microarray data; FR performed the integrative analysis; EC performed the *in vivo* experiments; MB and MG carried out sequencing experiments, MD and IA carried out microarray experiments; RD, FR, EC, and LP wrote the manuscript; GR, LP, and MP supervised the work; all authors read and approved the final manuscript.

## Conflict of Interest Statement

The authors declare that the research was conducted in the absence of any commercial or financial relationships that could be construed as a potential conflict of interest.

## Footnotes

^1^http://www.bioinformatics.babraham.ac.uk/projects/fastqc/

^2^http://www.bioconductor.com

^3^http://genome.ucsc.edu/

^4^http://www.ncbi.nlm.nih.gov/geo/query/acc.cgi?acc=GSE64466
