# Supplementary material for: Discovering miRNA Regulatory Networks in Holt–Oram Syndrome Using a Zebrafish Model
Source: Front Bioeng Biotechnol. 2016 Jul 14;4:60. doi: 10.3389/fbioe.2016.00060 (PMC4943955; doi:10.3389/fbioe.2016.00060)
Supplement: Supplementary file 1 [file Data_Sheet_1.ZIP › Tables.PDF]

Supplementary Material: Discovering miRNA regulatory networks in Holt-Oram Syndrome using a Zebrafish model

Romina D'Aurizio [1], Francesco Russo [1,2], Elena Chiavacci [3], Mario Baumgart[4], Marco Groth[4], Mara D'Onofrio[5], Ivan Arisi[5], Giuseppe Rainaldi [1], Letizia Pitto [3,*] and Marco Pellegrini [1,*]

[1] *Laboratory of Integrative Systems Medicine (LISM), Institute of Informatics and Telematics (IIT) and Institute of Clinical Physiology (IFC), National Research Council (CNR), Pisa, Italy*
[2] *Department of Computer Science, University of Pisa, Pisa, Italy*
[3] *Institute of Clinical Physiology (IFC), National Research Council (CNR), Pisa, Italy*
[4] *Leibniz Institute for Age Research, Fritz Lipmann Institute (FLI), Jena, Germany*
[5] *Genomics facility,Fondazione EBRI Rita Levi-Montalcini, Roma, Italy*

Correspondence*:
Marco Pellegrini
LISM-CNR, marco.pellegrini@iit.cnr.it

Letizia Pitto
Institute of Clinical Physiology (IFC), National Research Council (CNR), Pisa, 56124, Italy, l.pitto@ifc.cnr.it

Bioinformatics of Non-Coding RNAs with Applications to Biomedicine: Recent Advances and Open Challenges

1 SUPPLEMENTARY METHODS

Quantitative Real Time PCR.
cDNA was retro-transcribed using miRNA Reverse Transcription Kit: miScript II RT Kit-QIAGEN (for miRNA analysis) and Quantitec Reverse Transcription kit QIAGEN (for gene analysis). Real-time PCR (qRT-PCR) was carried out using SsoAdvanced Universal SYBR Green Supermix (Bio-Rad) with Rotor gene (Quiagen). EF1, βactin and 18S were used as internal standards for gene expression normalization, while U6 was used for miRNA normalization. Primer sequences are listed in table 1.All reactions were performed in triplicate.Relative quantification of gene expression was calculated as described Pfaffl et al. 2001.

Supplementary Table 1. Sequence of oligonucleotides used for qRT-PCR.

Gene	Forward primer	Reverse primer
EF1	CTGGAGGCCAGCTCAAACAT	ATCAAGAAGAGTAGTACCGCTAGCATTAC
βactin	CGAGCTGTCTTCCCATCC	TCACCAACGTAGCTGTCTTTCTG
18S	TCGCTAGTTGGCATCGTTTATG	CGGAGGTTCGAAGACGATCA
Hand2	AAGGCGAAAGAAGGAAATGAA	GCCAACCAGTTCTCCCTTTA
SRFa	AGCCGTTCCCCTTCATTC	GTGAGGGTGCTACTGCTGCT
ATP1a2a	CGACAACTTTGCTTCAATCG	TTAGGTTGTCGAAGATCAGACG
NDRG4	CCGTACTGACCCATCTTTTCA	TCTGCACAACTTCTGTGTTGC
MEF2AA	GGGGACCACGGAGAAAAA	TGGCTTTCAATGCCTTCTCT
CamK2d1	GATGGGAGTGGTCCATCGT	GCTGCACCCTTTAGTTTGCT
Up-1	-	TGAATCGAGCACCAGTTACGC
U6	ATGACACGCAAATCCGTGAAG	-
miR-7b	TGGAAGACTTGTGATTTTGTT	-
miR-10d-5p	TACCCTGTAGAACCGAATGTGTG	-
miR-19a-3p	TGTGCAAATCTATGCAAAACT	-
miR-21	TAGCTTATCAGACTGGTGTTGGC	-
miR-30a	TGTAAACATTCCCGACTGGAAG	-
miR-34a	TGGCAGTGTCTTAGCTGGTTGT	-
miR-210-5p	AGCCACTGACTAACGCACATTG	-
miR-210-3p	CTGTGCGTGTGACAGCGGCTAA	-
miR-219-1	TGATTGTCCAAACGCAATTCTT	-

2 SUPPLEMENTARY TABLES AND FIGURES

Supplementary Table 2. Statistics of sequencing experiments. Number of produced raw reads, trimmed reads and identified miRNAs are reported per sample.

Samples	Total Reads	Trimmed Reads (%)	miRNAs Reads	identified miRNAs
MO-Tbx5a.24hpf R1	16806257	14151564(84,20%)	708686	360
MO-Tbx5a.24hpf R2	18670675	14359051(76,91%)	1213272	369
MO-Ct.24hpf R1	25125923	20213514(80,45%)	858174	369
MO-Ct.24hpf R2	14305062	11220411(78,44%)	982681	366
MO-Tbx5a.48hpf	13410245	10583783(78,92%)	492152	372
MO-Ct.48hpf	12748255	11314491(88,75%)	338938	369

Supplementary Table 3. Modulated miRNAs between MO-Tbx5 and MO-Ct samples at 24hpf.

miRNAs	baseMean	FC	p-value	p-adj
dre-miR-34a	1024.59	2.82	1.03e-12	2.99e-10
dre-miR-16c	2943.61	1.49	1.67e-03	2.42e-02
dre-miR-205	24875.56	0.71	1.08e-03	1.96e-02
dre-miR-30d	15861.54	0.71	2.63e-03	3.19e-02
dre-miR-10a-5p	171332.67	0.71	3.72e-03	3.86e-02
dre-miR-182-5p	17628.21	0.71	2.48e-03	3.18e-02
dre-miR-206	3534.56	0.71	1.43e-03	2.40e-02
dre-miR-9-5p	2571.68	0.70	3.68e-03	3.86e-02
dre-miR-18c	1446.68	0.68	4.90e-03	4.64e-02
dre-miR-133a	2189.79	0.68	6.50e-04	1.29e-02
dre-miR-153c	604.96	0.65	2.79e-03	3.20e-02
dre-miR-204	2666.95	0.61	4.19e-05	1.31e-03
dre-miR-375	674.30	0.57	1.98e-04	5.39e-03
dre-miR-181c	1176.79	0.57	1.41e-05	6.15e-04
dre-miR-216b	446.31	0.57	2.29e-04	5.39e-03
dre-miR-10d-5p	26354.39	0.55	1.24e-07	6.77e-06
dre-miR-30a	3125.25	0.41	9.40e-12	1.02e-09
dre-miR-210-3p	356.71	0.33	8.29e-12	1.02e-09
dre-miR-210-5p	214.86	0.26	1.68e-10	1.22e-08

Supplementary Table 4. Modulated miRNAs between MO-Tbx5 and MO-Ct samples at 48hpf.

miRNAs	baseMean	FC	p-value	p-adj
dre-miR-34a	379.00	6.62	7.43e-16	2.70e-14
dre-miR-462	413.44	5.60	5.95e-10	7.63e-09
dre-miR-146a	419.40	4.50	1.05e-09	1.27e-08
dre-miR-430c	2583.79	4.13	4.36e-17	1.36e-15
dre-miR-430b	94853.04	3.16	1.60e-12	2.68e-11
dre-miR-430a	49675.09	3.14	1.98e-12	3.09e-11
dre-miR-21	33601.02	2.84	1.65e-10	2.25e-09
dre-miR-222a	13583.39	2.16	2.12e-06	1.85e-05
dre-miR-30a	3095.19	2.14	1.10e-05	8.26e-05
dre-miR-196b	770.91	2.00	7.45e-05	5.08e-04
dre-miR-203b-5p	1033.80	1.99	1.72e-04	1.07e-03
dre-miR-454b	14427.06	1.87	1.03e-04	6.84e-04
dre-miR-203b-3p	16393.40	1.86	1.31e-04	8.40e-04
dre-miR-203a	4218.27	1.80	3.78e-04	2.22e-03
dre-miR-206	2480.75	1.74	1.32e-03	6.84e-03
dre-miR-153c	2145.62	0.52	6.10e-04	3.33e-03
dre-miR-181a-3p	8254.53	0.47	3.87e-06	3.24e-05
dre-miR-153a	708.62	0.46	1.46e-03	7.40e-03
dre-let-7g	516.51	0.43	4.93e-04	2.78e-03
dre-miR-138	2454.87	0.42	5.51e-06	4.45e-05
dre-miR-182-5p	35595.61	0.41	2.99e-08	2.96e-07
dre-miR-125b	1358.40	0.41	3.25e-05	2.29e-04
dre-miR-183	5936.23	0.40	9.09e-08	8.62e-07
dre-miR-181c	6342.55	0.37	1.30e-08	1.35e-07
dre-miR-181b	26082.63	0.37	1.31e-09	1.43e-08
dre-miR-124	614.03	0.32	1.16e-05	8.42e-05
dre-miR-181a-5p	158025.61	0.31	9.09e-13	1.86e-11
dre-miR-2188-5p	328.52	0.28	2.00e-04	1.21e-03
dre-miR-9-5p	35945.98	0.24	9.18e-18	3.33e-16
dre-miR-125a	3086.02	0.21	1.12e-14	2.71e-13
dre-mir-7b	276.89	0.10	1.83e-07	1.66e-06
dre-miR-190b	321.28	0.01	1.21e-18	5.29e-17

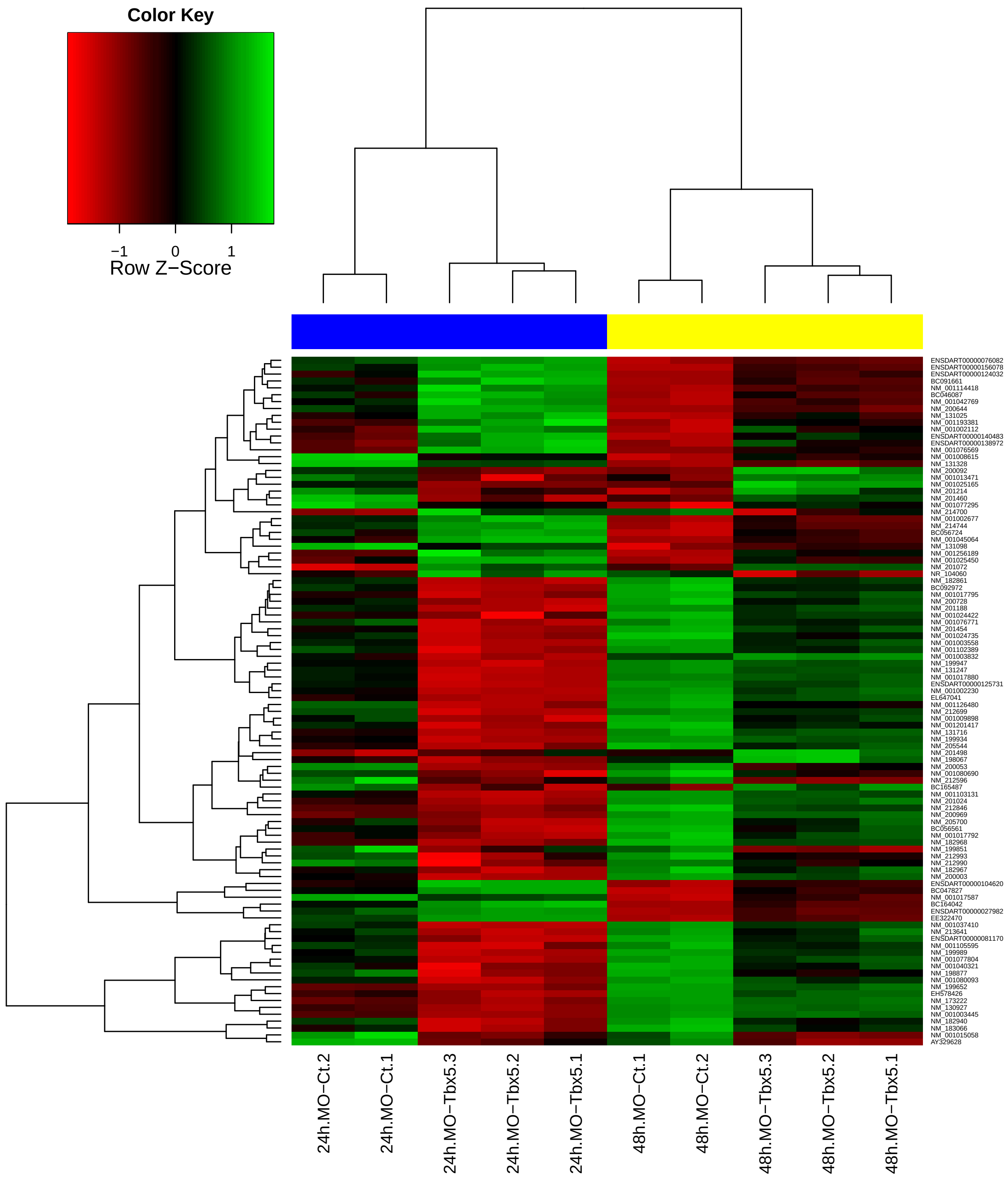

Figure 1. Heatmap plot of 100 most expressed and modulated genes both at 24 and 48 hpf.

Supplementary Table 5. Predicted interactions at 24hpf for upregulated miRNAs.

Target (RefSeq accession)	Gene symbol	miRNAs (miRBase ID)
NM_198067	Mmp2	dre-miR-34a
NM_001001843	tph1b	dre-miR-34a
NM_001002348	zgc:92174	dre-miR-34a
NM_001002354	bpnt1	dre-miR-34a
NM_001002357	zgc:92129	dre-miR-34a
NM_001002445	rhoad	dre-miR-34a
NM_001002449	zgc:92345	dre-miR-34a
NM_001002500	OTUB1	dre-miR-34a
NM_001003423	tnni2b.2	dre-miR-34a
NM_001003484	caprin1a	dre-miR-34a
NM_001003499	zgc:91985	dre-miR-34a
NM_001003587	glrbb	dre-miR-34a
NM_001003747	Ndufv1	dre-miR-34a
NM_001004121	cttn	dre-miR-34a
NM_001004580	zgc:92308	dre-miR-34a
NM_001004607	zgc:92599	dre-miR-34a
NM_001004642	ildr1	dre-miR-34a
NM_001005929	PAXIP1	dre-miR-34a
NM_001006085	zgc:101658	dre-miR-34a
NM_001006090	fam73a	dre-miR-34a
NM_001007207	t54l	dre-miR-34a
NM_001007288	cx30.9	dre-miR-34a
NM_001007317	zgc:92177	dre-miR-34a
NM_001007366	si:dkey-30j22.11	dre-miR-34a
NM_001007454	fut9	dre-miR-34a
NM_001007775	ptp4a1	dre-miR-34a
NM_001009987	zgc:86609	dre-miR-34a
NM_001009989	prkar1aa	dre-miR-34a
NM_001009990	Cdc40	dre-miR-34a
NM_001013259	serpina1l	dre-miR-34a
NM_001014292	hmgcrb	dre-miR-34a
NM_001017664	zgc:112054	dre-miR-34a
NM_001020520	MipB	dre-miR-34a
NM_001020672	vtna	dre-miR-34a
NM_001024405	Uchl3	dre-miR-34a
NM_001024414	zgc:112421	dre-miR-34a
NM_001024440	zgc:110417	dre-miR-34a
NM_001025517	Vps35	dre-miR-34a
NM_001029962	zgc:114085	dre-miR-34a
NM_001030143	Slc25a26	dre-miR-34a
NM_001030170	zgc:113381	dre-miR-34a
NM_001040337	zgc:136360	dre-miR-34a
NM_001040366	zgc:136850	dre-miR-34a
NM_001040394	UNC119B	dre-miR-34a
NM_001044352	WDR69	dre-miR-34a
NM_001044395	TAF9	dre-miR-34a
NM_001044850	TDRD5	dre-miR-34a
NM_001044862	si:dkey-12o15.1	dre-miR-34a
NM_001045485	Pi4k2b	dre-miR-34a
NM_001045847	NPRL2	dre-miR-34a
NM_001076612	zic4	dre-miR-34a
NM_001077758	SERPINA1	dre-miR-34a
NM_001079988	inka1a	dre-miR-34a
NM_001080572	SLC29A4	dre-miR-34a
NM_001080583	rdh10a	dre-miR-34a
NM_001080647	zgc:158644	dre-miR-34a
NM_001083547	DNAJC10	dre-miR-34a
NM_001089421	MAP2K4	dre-miR-34a
NM_001098386	wu:fc61g08	dre-miR-34a

Supplementary Table 5. continued...

Target (RefSeq accession)	Gene symbol	miRNAs (miRBase ID)
NM_001098487	selt2	dre-miR-34a
NM_001098740	SdhB	dre-miR-34a
NM_001105602	stx11b.2	dre-miR-34a
NM_001105683	ckmb	dre-miR-34a
NM_001109699	zgc:165580	dre-miR-34a
NM_001110371	ppp4r2b	dre-miR-34a
NM_001114442	ugt5g1	dre-miR-34a
NM_001115060	si:ch73-6k14.1	dre-miR-34a
NM_001126467	si:dkey-22o12.2	dre-miR-34a
NM_001128292	Prox2	dre-miR-34a
NM_001142774	gpt2l	dre-miR-34a
NM_001163293	SNX25	dre-miR-34a
NM_001168264	WDR37	dre-miR-34a
NM_130955	dld	dre-miR-34a
NM_131005	epd	dre-miR-34a
NM_131034	Gbas	dre-miR-34a
NM_131370	Acat2	dre-miR-34a
NM_131400	tef	dre-miR-34a
NM_131509	Krt4	dre-miR-34a
NM_131513	CCNB1	dre-miR-34a
NM_131558	zic2a	dre-miR-34a
NM_131667	gch2	dre-miR-34a
NM_131683	atp1a2a	dre-miR-34a
NM_152958	PHC2	dre-miR-34a
NM_153667	tpi1a	dre-miR-34a
NM_173231	CRYBB1	dre-miR-34a
NM_181559	glula	dre-miR-34a
NM_197934	RPN1	dre-miR-34a
NM_198366	eif4a1a	dre-miR-34a
NM_199544	zgc:77714	dre-miR-34a
NM_199547	ssb	dre-miR-34a
NM_199559	mier1a	dre-miR-34a
NM_199593	SHFM1	dre-miR-34a
NM_199643	coro2a	dre-miR-34a
NM_199688	GOSR2	dre-miR-34a
NM_199850	c20orf24	dre-miR-34a
NM_199868	HGS	dre-miR-34a
NM_200000	zgc:77231	dre-miR-34a
NM_200174	zgc:55702	dre-miR-34a
NM_200242	zgc:56041	dre-miR-34a
NM_200251	slc43a2a	dre-miR-34a
NM_200649	acsl4a	dre-miR-34a
NM_200928	Rab7	dre-miR-34a
NM_205558	Ap1s2	dre-miR-34a
NM_205644	insm1a	dre-miR-34a
NM_205663	zgc:77456	dre-miR-34a
NM_205700	Cfl2	dre-miR-34a
NM_212439	per1b	dre-miR-34a
NM_212641	pa2g4b	dre-miR-34a
NM_212652	SLC35B4	dre-miR-34a
NM_212663	CD82	dre-miR-34a
NM_212695	kdelr2l	dre-miR-34a
NM_212722	eno1	dre-miR-34a
NM_212742	Myst1	dre-miR-34a
NM_212779	mpx	dre-miR-34a
NM_212866	zgc:77051	dre-miR-34a
NM_212886	pgm2	dre-miR-34a
NM_212894	lgals2a	dre-miR-34a
NM_212898	mettl11a	dre-miR-34a

Supplementary Table 5. continued...

Target (RefSeq accession)	Gene symbol	miRNAs (miRBase ID)
NM_213181	ptp4a3	dre-miR-34a
NM_213336	ctsba	dre-miR-34a
NM_213375	VPS26B	dre-miR-34a
NM_213444	Derl1	dre-miR-34a

Supplementary Table 6. Predicted interactions at 24hpf for downregulated miRNAs.

Target (RefSeq accession)	Gene symbol	miRNAs (miRBase ID)
NM_001001825	zgc:77123	dre-miR-30a-5p
NM_001001844	CTCF	dre-miR-30a-5p
NM_001002044	mafk	dre-miR-210-5p
NM_001002112	zgc:86833	dre-miR-210-3p
NM_001002180	MGAT4B	dre-miR-10d-5p,dre-miR-210-5p
NM_001002305	zgc:92022	dre-miR-210-3p
NM_001002319	UBLCP1,wu:fb33g09	dre-miR-30a-5p
NM_001002492	CMTM4	dre-miR-10d-5p
NM_001002626	zgc:92242	dre-miR-30a-5p
NM_001002656	DGCR2	dre-miR-210-5p
NM_001002660	sesn1	dre-miR-210-5p,dre-miR-30a-5p
NM_001002667	slc25a36a	dre-miR-210-3p,dre-miR-210-5p,dre-miR-30a-5p
NM_001002738	KCTD9	dre-miR-30a-5p
NM_001003478	prr15la	dre-miR-30a-5p
NM_001003498	zgc:91999	dre-miR-210-5p
NM_001003518	CHST1	dre-miR-210-3p,dre-miR-30a-5p
NM_001003538	zgc:100849	dre-miR-210-5p
NM_001003602	camk2d1	dre-miR-30a-5p
NM_001003606	FAM49A	dre-miR-30a-5p
NM_001003739	RAD23A	dre-miR-30a-5p
NM_001003751	zgc:100913	dre-miR-10d-5p,dre-miR-30a-5p
NM_001003780	YPEL1	dre-miR-210-3p,dre-miR-210-5p
NM_001003833	si:dkeyp-84g9.1	dre-miR-30a-5p
NM_001003981	PTN	dre-miR-210-3p
NM_001004628	pnp5b	dre-miR-30a-5p
NM_001004648	zgc:101062	dre-miR-210-3p
NM_001005401	SRP68	dre-miR-30a-5p
NM_001005591	zgc:100918	dre-miR-210-3p
NM_001005966	zgc:101848	dre-miR-210-3p,dre-miR-210-5p,dre-miR-30a-5p
NM_001005974	zgc:103601	dre-miR-30a-5p
NM_001006026	Csrp3	dre-miR-210-5p
NM_001006028	fhl2b	dre-miR-30a-5p
NM_001006035	zgc:103440	dre-miR-30a-5p
NM_001007051	vat1	dre-miR-210-3p,dre-miR-30a-5p
NM_001007330	cbl	dre-miR-10d-5p,dre-miR-210-5p,dre-miR-30a-5p
NM_001007359	Rab11a	dre-miR-30a-5p
NM_001007369	STAM2	dre-miR-210-5p
NM_001007405	RBM41	dre-miR-210-5p
NM_001007410	YTHDC1	dre-miR-30a-5p
NM_001007432	Galk2	dre-miR-30a-5p
NM_001008605	zgc:103508	dre-miR-210-5p
NM_001008643	zgc:101644	dre-miR-210-5p
NM_001008644	bmp2k	dre-miR-30a-5p
NM_001009914	atg5	dre-miR-210-5p
NM_001012373	rybpb	dre-miR-30a-5p
NM_001012487	zgc:113026	dre-miR-30a-5p
NM_001013333	zgc:110796	dre-miR-30a-5p
NM_001013489	zgc:113336	dre-miR-30a-5p
NM_001013521	Enah,zgc:103638	dre-miR-30a-5p
NM_001014303	ERLIN1	dre-miR-210-5p
NM_001014305	zgc:110843	dre-miR-10d-5p,dre-miR-210-3p
NM_001014306	lifra	dre-miR-30a-5p
NM_001014314	zgc:113259	dre-miR-10d-5p
NM_001014337	zgc:113176	dre-miR-210-3p,dre-miR-30a-5p
NM_001014363	ablim1b	dre-miR-30a-5p
NM_001017596	zgc:110298	dre-miR-30a-5p
NM_001017665	zgc:112052	dre-miR-10d-5p,dre-miR-210-3p,dre-miR-30a-5p
NM_001017670	ms4a17a.6	dre-miR-210-5p
NM_001017749	arl4ca	dre-miR-210-5p,dre-miR-30a-5p

Supplementary Table 6. continued...

Target (RefSeq accession)	Gene symbol	miRNAs (miRBase ID)
NM_001017813	zgc:110314	dre-miR-210-5p,dre-miR-30a-5p
NM_001018140	dnmt6	dre-miR-30a-5p
NM_001018147	v2rd1	dre-miR-30a-5p
NM_001020490	ezrl	dre-miR-10d-5p
NM_001020591	Syngr3	dre-miR-30a-5p
NM_001020612	gmpr	dre-miR-10d-5p
NM_001020618	zgc:110655	dre-miR-210-3p
NM_001020629	hrasb	dre-miR-30a-5p
NM_001020671	zgc:112270	dre-miR-30a-5p
NM_001020679	zgc:112317	dre-miR-30a-5p
NM_001020702	FBXO3	dre-miR-210-3p
NM_001020735	pcyt1ab	dre-miR-30a-5p
NM_001023581	tmem167a	dre-miR-30a-5p
NM_001024426	zgc:110022	dre-miR-210-3p,dre-miR-30a-5p
NM_001024653	KDR	dre-miR-30a-5p
NM_001024815	BTBD6	dre-miR-10d-5p
NM_001025450	dnmt4	dre-miR-30a-5p
NM_001025527	si:ch211-239e6.4	dre-miR-30a-5p
NM_001025538	si:dkey-24l11.4	dre-miR-10d-5p
NM_001029958	si:dkey-30h14.2	dre-miR-30a-5p
NM_001030125	camk2g1	dre-miR-30a-5p
NM_001030175	zgc:110105	dre-miR-210-3p,dre-miR-210-5p
NM_001030193	zgc:114081	dre-miR-10d-5p
NM_001030206	zgc:114140	dre-miR-10d-5p,dre-miR-30a-5p
NM_001030245	Fbxl18	dre-miR-30a-5p
NM_001033591	zgc:112334	dre-miR-10d-5p
NM_001037243	Lhx9	dre-miR-10d-5p
NM_001037661	slc6a6	dre-miR-30a-5p
NM_001039817	bmpr2a	dre-miR-10d-5p,dre-miR-30a-5p
NM_001040035	AARS	dre-miR-30a-5p
NM_001040313	zgc:136739	dre-miR-10d-5p,dre-miR-30a-5p
NM_001040332	si:ch211-11m18.3	dre-miR-210-5p
NM_001040341	ATXN7L3	dre-miR-30a-5p
NM_001040391	BCL11A	dre-miR-210-5p
NM_001040393	zgc:136817	dre-miR-30a-5p
NM_001044326	zmynd11	dre-miR-30a-5p
NM_001044333	si:ch211-67n3.1	dre-miR-10d-5p
NM_001044778	poc1bl	dre-miR-210-5p
NM_001044791	CD164	dre-miR-210-5p
NM_001044828	zgc:162777	dre-miR-10d-5p,dre-miR-210-3p
NM_001044830	zgc:153031	dre-miR-210-5p
NM_001044833	dyrk2	dre-miR-30a-5p
NM_001044834	si:dkey-239i20.4	dre-miR-10d-5p,dre-miR-30a-5p
NM_001044865	CHKA	dre-miR-30a-5p
NM_001044879	si:ch211-81a5.7	dre-miR-30a-5p
NM_001044918	si:ch211-203l9.3	dre-miR-10d-5p
NM_001044988	si:dkey-72l14.4	dre-miR-210-3p
NM_001045064	hcfc1a	dre-miR-30a-5p
NM_001045158	SBF1	dre-miR-30a-5p
NM_001045175	KCNJ1	dre-miR-30a-5p
NM_001045241	si:ch211-160d20.3	dre-miR-30a-5p
NM_001045322	crb3a	dre-miR-210-5p
NM_001045369	zgc:153184	dre-miR-30a-5p
NM_001045439	Hnrnpa3	dre-miR-30a-5p
NM_001045846	zgc:153966	dre-miR-30a-5p
NM_001048235	srgap2	dre-miR-30a-5p
NM_001076615	ctdsplb	dre-miR-210-3p
NM_001076653	srd5a1	dre-miR-30a-5p
NM_001076718	adam28	dre-miR-210-5p

Supplementary Table 6. continued...

Target (RefSeq accession)	Gene symbol	miRNAs (miRBase ID)
NM_001077147	zgc:153606	dre-miR-30a-5p
NM_001077276	zgc:153948	dre-miR-210-5p
NM_001077288	zgc:152938	dre-miR-10d-5p
NM_001077382	zgc:153923	dre-miR-30a-5p
NM_001077432	si:ch211-57i17.1	dre-miR-210-3p,dre-miR-30a-5p
NM_001077451	CCDC120,si:dkey-13a21.14	dre-miR-210-5p,dre-miR-30a-5p
NM_001077570	scn4ba	dre-miR-30a-5p
NM_001077626	crfb2	dre-miR-10d-5p
NM_001077726	zgc:154064	dre-miR-210-3p,dre-miR-30a-5p
NM_001077756	zgc:153892	dre-miR-30a-5p
NM_001077766	FAM160B2	dre-miR-210-5p
NM_001079955	DIDO1	dre-miR-210-5p
NM_001079962	NFIA	dre-miR-30a-5p
NM_001079969	spopla,spoplb	dre-miR-210-3p,dre-miR-210-5p,dre-miR-30a-5p
NM_001080029	arrdc3	dre-miR-10d-5p
NM_001080047	nrxn3b	dre-miR-210-3p,dre-miR-30a-5p
NM_001080167	si:ch211-234g24.1	dre-miR-210-3p,dre-miR-30a-5p
NM_001080169	ubn2	dre-miR-30a-5p
NM_001080172	zgc:158663	dre-miR-30a-5p
NM_001080188	git2b	dre-miR-210-5p
NM_001080588	zgc:153949	dre-miR-210-5p
NM_001080606	zgc:158667	dre-miR-30a-5p
NM_001080659	zgc:158689	dre-miR-210-3p
NM_001080662	zgc:158294	dre-miR-210-3p
NM_001080683	zgc:158647	dre-miR-10d-5p,dre-miR-30a-5p
NM_001080750	Sox7	dre-miR-30a-5p
NM_001082805	KDM4B	dre-miR-30a-5p
NM_001082808	PXK	dre-miR-30a-5p
NM_001082810	si:ch211-244b2.2,si:ch211-244b2.3	dre-miR-30a-5p
NM_001082997	si:dkey-23c22.6	dre-miR-210-5p,dre-miR-30a-5p
NM_001083575	eomesb	dre-miR-10d-5p
NM_001083812	ELL2	dre-miR-30a-5p
NM_001083817	si:ch211-182e10.4	dre-miR-30a-5p
NM_001089326	ctdspl2b	dre-miR-30a-5p
NM_001089329	pou2f1b	dre-miR-30a-5p
NM_001089337	zgc:158623	dre-miR-210-5p
NM_001089376	si:rp71-10d23.3	dre-miR-210-5p
NM_001089417	pfkmb	dre-miR-30a-5p
NM_001089427	zgc:162879	dre-miR-30a-5p
NM_001089554	si:dkey-251i10.1	dre-miR-210-5p
NM_001089575	si:dkey-4c15.14	dre-miR-30a-5p
NM_001098765	zgc:165543	dre-miR-210-5p
NM_001098776	zgc:165631	dre-miR-210-5p
NM_001099246	KIF19,abca5	dre-miR-210-3p
NM_001099259	RAB8B	dre-miR-30a-5p
NM_001099449	Tmem2	dre-miR-210-5p,dre-miR-30a-5p
NM_001099973	vps33a	dre-miR-30a-5p
NM_001100441	zgc:165621	dre-miR-30a-5p
NM_001100957	HSPB8	dre-miR-30a-5p
NM_001109727	si:dkey-263h23.4	dre-miR-30a-5p
NM_001110200	gxylt2	dre-miR-30a-5p
NM_001110396	snx10b	dre-miR-210-3p
NM_001110456	oxnad1	dre-miR-210-3p,dre-miR-210-5p
NM_001110521	LOC563864	dre-miR-10d-5p
NM_001111089	LOC794719,si:dkey-20i20.2	dre-miR-30a-5p
NM_001111169	DPF3	dre-miR-210-3p
NM_001111193	zgc:171495	dre-miR-30a-5p
NM_001113507	IL7R	dre-miR-210-3p,dre-miR-30a-5p
NM_001113590	zgc:172115	dre-miR-210-5p

Supplementary Table 6. continued...

Target (RefSeq accession)	Gene symbol	miRNAs (miRBase ID)
NM_001113611	zgc:171813	dre-miR-30a-5p
NM_001113618	Mlec	dre-miR-210-3p
NM_001113638	zgc:171551	dre-miR-30a-5p
NM_001114575	si:ch211-95o16.2	dre-miR-30a-5p
NM_001114584	zgc:172295	dre-miR-210-5p
NM_001114707	si:ch211-157p22.10	dre-miR-210-3p,dre-miR-30a-5p
NM_001114742	zgc:171544	dre-miR-30a-5p
NM_001114908	zgc:171818	dre-miR-210-5p
NM_001114911	zgc:172265	dre-miR-30a-5p
NM_001114929	si:ch211-244b2.2	dre-miR-30a-5p
NM_001122613	sc:d158	dre-miR-30a-5p
NM_001122624	zgc:175247	dre-miR-30a-5p
NM_001122749	Dgcr8	dre-miR-210-5p,dre-miR-30a-5p
NM_001122844	CUL4B	dre-miR-10d-5p,dre-miR-30a-5p
NM_001123012	aplp2	dre-miR-210-5p
NM_001123045	zgc:158284	dre-miR-210-3p,dre-miR-30a-5p
NM_001123055	zgc:158420	dre-miR-30a-5p
NM_001123058	zgc:171814	dre-miR-10d-5p,dre-miR-210-5p,dre-miR-30a-5p
NM_001123302	zgc:113363	dre-miR-210-5p
NM_001126423	ddx3	dre-miR-30a-5p
NM_001126448	LECT1	dre-miR-30a-5p
NM_001126449	pdxka	dre-miR-30a-5p
NM_001126460	si:dkey-169i5.4	dre-miR-10d-5p
NM_001127369	Npb	dre-miR-30a-5p
NM_001127473	zgc:175146	dre-miR-210-5p
NM_001128242	atp2b3b	dre-miR-10d-5p
NM_001128266	LOC792835	dre-miR-30a-5p
NM_001128270	si:busm1-112p11.1	dre-miR-210-3p,dre-miR-30a-5p
NM_001128298	zgc:173506	dre-miR-30a-5p
NM_001128329	lypd6b	dre-miR-210-5p,dre-miR-30a-5p
NM_001128355	si:ch211-205a14.7	dre-miR-30a-5p
NM_001128529	Mepce	dre-miR-30a-5p
NM_001128721	si:dkey-63j12.4	dre-miR-10d-5p
NM_001130402	si:ch211-195h23.3	dre-miR-10d-5p
NM_001135104	zeb2a	dre-miR-30a-5p
NM_001135108	CAPN7	dre-miR-10d-5p,dre-miR-30a-5p
NM_001135784	nkx6.2	dre-miR-10d-5p
NM_001136477	fam125bb	dre-miR-30a-5p
NM_001144045	KIFAP3	dre-miR-10d-5p
NM_001144784	inaA	dre-miR-30a-5p
NM_001145579	si:ch211-198a12.6	dre-miR-30a-5p
NM_001145589	si:ch211-206k20.4	dre-miR-210-5p
NM_001159833	zgc:136867	dre-miR-30a-5p
NM_001160126	LOC100149189,si:dkeyp-22b2.3	dre-miR-210-5p
NM_001161670	mbnl2	dre-miR-30a-5p
NM_001161671	mbnl2	dre-miR-30a-5p
NM_001163298	si:dkey-223d7.5	dre-miR-30a-5p
NM_130916	inhbaa	dre-miR-30a-5p
NM_130944	dlc	dre-miR-30a-5p
NM_130968	Odz3	dre-miR-30a-5p
NM_131026	efna5b	dre-miR-30a-5p
NM_131037	fsta	dre-miR-210-5p,dre-miR-30a-5p
NM_131044	eng2a	dre-miR-10d-5p,dre-miR-30a-5p
NM_131160	brn1.2,pou3f2	dre-miR-210-5p,dre-miR-30a-5p
NM_131250	otx1b	dre-miR-210-3p
NM_131275	rxrba	dre-miR-10d-5p
NM_131299	FOXA3	dre-miR-30a-5p
NM_131312	mef2ca	dre-miR-10d-5p
NM_131317	MEF2D	dre-miR-210-3p

Supplementary Table 6. continued...

Target (RefSeq accession)	Gene symbol	miRNAs (miRBase ID)
NM_131324	LOC100149335,pax7a	dre-miR-210-3p,dre-miR-210-5p,dre-miR-30a-5p
NM_131354	six7	dre-miR-210-5p
NM_131374	PSME2	dre-miR-30a-5p
NM_131379	kal1b	dre-miR-30a-5p
NM_131481	ROBO1	dre-miR-30a-5p
NM_131503	AXIN1	dre-miR-30a-5p
NM_131517	spon1b	dre-miR-30a-5p
NM_131539	Pim1	dre-miR-30a-5p
NM_131556	fgd	dre-miR-30a-5p
NM_131628	scn8aa	dre-miR-30a-5p
NM_131674	ARNT2	dre-miR-210-3p
NM_131719	cdk5	dre-miR-30a-5p
NM_131736	SLIT3	dre-miR-30a-5p
NM_131741	or115-14	dre-miR-30a-5p
NM_131769	cldnj	dre-miR-30a-5p
NM_131773	cldn12	dre-miR-30a-5p
NM_131858	SFRP5	dre-miR-10d-5p,dre-miR-210-5p
NM_131862	JAG2	dre-miR-210-3p,dre-miR-210-5p,dre-miR-30a-5p
NM_131870	GUCA1A	dre-miR-30a-5p
NM_131888	PTPRA	dre-miR-210-3p
NM_131893	MEIS1	dre-miR-210-5p
NM_153652	PLAGL2	dre-miR-210-3p,dre-miR-30a-5p
NM_153659	sec61al1	dre-miR-210-3p,dre-miR-210-5p
NM_178296	GLI1	dre-miR-10d-5p
NM_178303	FGFR2	dre-miR-210-3p
NM_180973	SP5	dre-miR-210-5p
NM_182884	DIXDC1	dre-miR-30a-5p
NM_182889	nfe2l2	dre-miR-210-5p
NM_194364	adra2da	dre-miR-30a-5p
NM_194410	VAX1	dre-miR-10d-5p
NM_198874	Lmbr1l	dre-miR-30a-5p
NM_198878	rbpja	dre-miR-10d-5p
NM_199209	Cbfb	dre-miR-30a-5p
NM_199522	rnd3a	dre-miR-30a-5p
NM_199530	SEC31A	dre-miR-30a-5p
NM_199536	rtcd1	dre-miR-210-3p
NM_199558	zfr	dre-miR-210-3p
NM_199633	WSB1	dre-miR-210-3p,dre-miR-210-5p,dre-miR-30a-5p
NM_199641	prkcbp1l	dre-miR-30a-5p
NM_199646	itm2ba	dre-miR-10d-5p
NM_199649	zfp36l1l	dre-miR-210-5p,dre-miR-30a-5p
NM_199669	polr2gl	dre-miR-210-5p
NM_199703	tnika	dre-miR-210-5p
NM_199758	ARIH1	dre-miR-210-5p
NM_199780	gats	dre-miR-30a-5p
NM_199828	CCNT2	dre-miR-210-5p,dre-miR-30a-5p
NM_199852	TAF5L	dre-miR-30a-5p
NM_199885	Mark3	dre-miR-30a-5p
NM_199898	BRF1	dre-miR-210-3p,dre-miR-30a-5p
NM_199926	tada3l	dre-miR-10d-5p,dre-miR-210-5p
NM_200099	dynll2a,dynll2b	dre-miR-30a-5p
NM_200107	zgc:64114	dre-miR-30a-5p
NM_200121	Tceb3	dre-miR-30a-5p
NM_200164	slc25a14	dre-miR-210-5p,dre-miR-30a-5p
NM_200217	zgc:56235	dre-miR-210-5p
NM_200276	NCOR1	dre-miR-10d-5p,dre-miR-210-5p,dre-miR-30a-5p
NM_200297	MGLL	dre-miR-10d-5p
NM_200358	SLC10A2	dre-miR-30a-5p
NM_200379	zgc:64119	dre-miR-210-3p

Supplementary Table 6. continued...

Target (RefSeq accession)	Gene symbol	miRNAs (miRBase ID)
NM_200385	GORAB	dre-miR-210-5p
NM_200454	zgc:63553	dre-miR-10d-5p,dre-miR-30a-5p
NM_200515	zgc:66285	dre-miR-30a-5p
NM_200561	FZD6	dre-miR-10d-5p
NM_200597	efna1a	dre-miR-10d-5p,dre-miR-210-5p,dre-miR-30a-5p
NM_200643	SLC30A4	dre-miR-210-3p,dre-miR-30a-5p
NM_200656	guca1e	dre-miR-210-3p
NM_200682	zgc:65888	dre-miR-30a-5p
NM_200753	zgc:73220	dre-miR-30a-5p
NM_200831	ampH	dre-miR-30a-5p
NM_200872	BECN1	dre-miR-30a-5p
NM_200892	ERP44	dre-miR-210-3p
NM_200893	AMPD1	dre-miR-30a-5p
NM_200923	vldlr	dre-miR-10d-5p
NM_200963	FH	dre-miR-210-3p
NM_200970	rab5ab	dre-miR-30a-5p
NM_200997	nck2a	dre-miR-10d-5p,dre-miR-30a-5p
NM_201001	ARL2BP	dre-miR-210-5p
NM_201072	RTN3	dre-miR-30a-5p
NM_201087	TNPO3	dre-miR-210-5p
NM_201130	SPOP	dre-miR-30a-5p
NM_201145	zgc:55746	dre-miR-30a-5p
NM_201168	sfrs8	dre-miR-210-3p
NM_201205	Ppih	dre-miR-30a-5p
NM_201461	copeb	dre-miR-210-5p
NM_201474	HDLBP	dre-miR-210-5p
NM_201492	TPM3	dre-miR-210-5p
NM_201502	SMOX	dre-miR-210-5p
NM_205547	Scamp5	dre-miR-10d-5p
NM_205586	PRNP	dre-miR-30a-5p
NM_205628	dmrt1	dre-miR-30a-5p
NM_205633	evlb	dre-miR-210-5p
NM_205665	zgc:77396	dre-miR-30a-5p
NM_205733	RABL3	dre-miR-30a-5p
NM_205761	dmrt1	dre-miR-10d-5p,dre-miR-30a-5p
NM_207049	KCTD5	dre-miR-30a-5p
NM_207052	MIDN	dre-miR-210-3p,dre-miR-30a-5p
NM_212561	HEY1	dre-miR-10d-5p
NM_212611	ACADVL	dre-miR-30a-5p
NM_212651	cav1	dre-miR-30a-5p
NM_212723	mknk2a	dre-miR-30a-5p
NM_212732	MDM4	dre-miR-210-3p
NM_212739	Fbxo42	dre-miR-30a-5p
NM_212754	Polr3e	dre-miR-30a-5p
NM_212781	yrk	dre-miR-10d-5p
NM_212804	Insig2	dre-miR-210-3p
NM_212834	nr5a1b	dre-miR-210-5p
NM_212836	cdx1a	dre-miR-210-3p,dre-miR-30a-5p
NM_212869	TRIB3	dre-miR-30a-5p
NM_212876	CYB5B	dre-miR-30a-5p
NM_212895	rbp1b	dre-miR-30a-5p
NM_212929	zgc:85680	dre-miR-10d-5p,dre-miR-30a-5p
NM_212941	mafg2	dre-miR-210-3p,dre-miR-30a-5p
NM_212966	nrp2b	dre-miR-30a-5p
NM_212987	UBE2Q2	dre-miR-30a-5p
NM_213001	KLHL20	dre-miR-30a-5p
NM_213062	Uba1	dre-miR-210-3p
NM_213064	zgc:55943	dre-miR-30a-5p
NM_213119	slc25a28	dre-miR-210-3p,dre-miR-30a-5p

Supplementary Table 6. continued...

Target (RefSeq accession)	Gene symbol	miRNAs (miRBase ID)
NM_213232	spred1	dre-miR-210-3p
NM_213280	RSRC2	dre-miR-30a-5p
NM_213342	ddc	dre-miR-210-5p
NM_213348	ndrg1	dre-miR-210-3p
NM_213357	gab1	dre-miR-210-3p,dre-miR-30a-5p
NM_213362	epn1	dre-miR-210-5p
NM_213364	p4hb	dre-miR-10d-5p
NM_213397	pfkfb3	dre-miR-10d-5p,dre-miR-30a-5p
NM_213408	SLC25A22	dre-miR-210-3p,dre-miR-30a-5p
NM_213432	e2f4	dre-miR-30a-5p
NM_213505	pob	dre-miR-210-5p
NM_213506	zgc:63491	dre-miR-10d-5p
NM_213549	matn4	dre-miR-10d-5p,dre-miR-30a-5p
NM_213637	Scrt2	dre-miR-30a-5p
NM_214692	MPP1	dre-miR-210-3p,dre-miR-30a-5p
NM_214750	zgc:65779	dre-miR-210-3p
NM_214772	zgc:77202	dre-miR-30a-5p
NM_214801	GPN2	dre-miR-30a-5p

Supplementary Table 7. Predicted interactions at 48hpf for upregulated miRNAs.

Target (RefSeq accession)	Gene Symbol	miRNAs (miRBase ID)
NM_001002212	LOC100149152,PRSS35	dre-miR-21
NM_001002348	zgc:92174	dre-miR-34a
NM_001002448	ING2	dre-miR-34a
NM_001002608	rgp1	dre-miR-21
NM_001002630	BPGM	dre-miR-146a,dre-miR-21
NM_001002733	nxph1	dre-miR-146a,dre-miR-34a
NM_001003423	tnni2b.2	dre-miR-34a
NM_001003516	zgc:100908	dre-miR-34a
NM_001003587	glrbb	dre-miR-34a
NM_001003764	DEXI	dre-miR-21
NM_001004502	lpar1	dre-miR-146a,dre-miR-34a
NM_001004545	mpdu1a	dre-miR-34a
NM_001004570	ccdc85al	dre-miR-21
NM_001004645	LCMT1	dre-miR-21
NM_001005777	apoob	dre-miR-21
NM_001006099	Baz2a	dre-miR-146a
NM_001007310	cyp1d1	dre-miR-21
NM_001007366	si:dkey-30j22.11	dre-miR-21,dre-miR-34a
NM_001007368	nr2e3	dre-miR-34a
NM_001007454	fut9,zgc:103510	dre-miR-34a
NM_001008607	hsdl1	dre-miR-21
NM_001009903	zgc:86586	dre-miR-21
NM_001013350	Agtr2	dre-miR-21
NM_001015063	snx18b	dre-miR-34a
NM_001017565	zgc:110552	dre-miR-146a
NM_001017598	sostdc1a	dre-miR-146a
NM_001020610	cyp1c1	dre-miR-21
NM_001020668	zgc:112242	dre-miR-21
NM_001020672	vtna	dre-miR-34a
NM_001024405	Uchl3	dre-miR-34a
NM_001024414	zgc:112421	dre-miR-34a
NM_001024415	cbx8b	dre-miR-146a
NM_001024423	crygmxl1	dre-miR-146a
NM_001025546	zgc:114196	dre-miR-34a
NM_001030253	BCL2	dre-miR-34a
NM_001033751	zgc:112435	dre-miR-21
NM_001037239	zgc:123177	dre-miR-146a
NM_001040321	Cirbp,LOC100002393,eef1da	dre-miR-21
NM_001044318	GNG2	dre-miR-146a
NM_001044395	TAF9	dre-miR-21,dre-miR-34a
NM_001044717	si:ch211-212d10.2	dre-miR-462
NM_001044870	si:dkey-42i9.6	dre-miR-146a
NM_001045014	si:ch211-106n13.3	dre-miR-34a
NM_001045173	ndrg4	dre-miR-21
NM_001045401	zgc:153411	dre-miR-21
NM_001045431	zgc:153708	dre-miR-21
NM_001045435	zgc:153612	dre-miR-146a
NM_001076673	zgc:153098	dre-miR-21
NM_001076775	TRAPPC2	dre-miR-146a,dre-miR-21
NM_001077463	zgc:153665	dre-miR-146a
NM_001077629	SCN2B	dre-miR-34a
NM_001077765	BMP3,si:ch211-57h10.1	dre-miR-146a,dre-miR-21
NM_001077767	papss2a	dre-miR-34a
NM_001077779	npsn,npsnl	dre-miR-146a
NM_001079960	mxra8b	dre-miR-146a
NM_001080197	si:ch211-258l4.3	dre-miR-462
NM_001080686	TMEM205	dre-miR-21
NM_001083861	zgc:162334	dre-miR-21
NM_001089334	zgc:162335	dre-miR-34a

Supplementary Table 7. continued...

Target (RefSeq accession)	Gene Symbol	miRNAs (miRBase ID)
NM_001098764	zgc:165481	dre-miR-146a,dre-miR-21
NM_001099740	MATN1	dre-miR-34a
NM_001100030	si:ch211-69i14.7	dre-miR-34a
NM_001111175	zgc:171566	dre-miR-146a
NM_001114434	GPR27	dre-miR-34a
NM_001114589	zgc:174910	dre-miR-21
NM_001126467	si:dkey-22o12.2	dre-miR-146a,dre-miR-34a
NM_001128335	si:ch211-215f19.1	dre-miR-21
NM_001145700	ATL1	dre-miR-146a
NM_001145782	col5a2l,si:busm1-167c3.3	dre-miR-21
NM_001159664	ZWI	dre-miR-34a
NM_001159834	zgc:153700	dre-miR-21
NM_001159984	si:ch211-210h11.4	dre-miR-146a
NM_130912	cat	dre-miR-146a
NM_130927	nme2b.2	dre-miR-146a
NM_131005	epd	dre-miR-34a
NM_131034	Gbas	dre-miR-34a
NM_131045	eng1a	dre-miR-21
NM_131120	hoxb8a	dre-miR-146a
NM_131193	Eya1	dre-miR-21
NM_131216	lhx1a	dre-miR-146a,dre-miR-462
NM_131323	dlx6a	dre-miR-146a
NM_131370	Acat2	dre-miR-34a
NM_131381	GSK3B	dre-miR-462
NM_131458	igfbp2a	dre-miR-21
NM_131499	sreb2	dre-miR-21
NM_131537	hoxb5b	dre-miR-146a
NM_131604	runx3	dre-miR-146a,dre-miR-34a
NM_131682	alaS2	dre-miR-34a
NM_131690	atp1a1b	dre-miR-146a
NM_131868	GNAT1	dre-miR-21
NM_153644	H2AFV,H2AFZ,h2afvl	dre-miR-21
NM_153667	tpi1a	dre-miR-34a
NM_173254	ATP6V1E1	dre-miR-462
NM_174861	GBX1	dre-miR-21
NM_175084	epb41	dre-miR-34a
NM_198071	prom1b	dre-miR-146a
NM_198877	mibp2	dre-miR-146a
NM_198981	Sprn	dre-miR-21
NM_199517	ndrg3a	dre-miR-146a
NM_199604	MAP1LC3B	dre-miR-21
NM_199625	socs6b	dre-miR-21
NM_199627	lgi1a	dre-miR-146a
NM_199786	Clec14a	dre-miR-34a
NM_199847	fxyd6	dre-miR-21
NM_199851	GTPBP4	dre-miR-146a
NM_199962	Rgs5	dre-miR-146a
NM_199963	rogdi	dre-miR-21
NM_200003	h3f3a,h3f3c,zgc:110292,zgc:56418	dre-miR-146a,dre-miR-21
NM_200009	zgc:73138	dre-miR-146a,dre-miR-34a
NM_200125	LOC566487,gbgt1l4	dre-miR-21
NM_200195	zgc:56112	dre-miR-462
NM_200248	POLM	dre-miR-462
NM_200559	zgc:66109	dre-miR-146a
NM_200692	ndrg1	dre-miR-21
NM_200700	pppde2a	dre-miR-146a
NM_200706	cib2	dre-miR-146a,dre-miR-462
NM_200870	znrf1	dre-miR-21
NM_200943	BAT1	dre-miR-146a

Supplementary Table 7. continued...

Target (RefSeq accession)	Gene Symbol	miRNAs (miRBase ID)
NM_201166	DCTN2	dre-miR-146a
NM_201291	id2a	dre-miR-146a
NM_201454	RAB2A	dre-miR-146a
NM_201471	aldh9a1a	dre-miR-462
NM_203427	gpm6ba	dre-miR-146a
NM_203460	adh8a,adh8b	dre-miR-21
NM_203461	CHM	dre-miR-146a
NM_205539	LRRC17	dre-miR-21
NM_205700	Cfl2	dre-miR-34a
NM_207083	zgc:76877	dre-miR-21
NM_212625	ST6GALNAC3	dre-miR-146a
NM_212716	smarca2	dre-miR-21
NM_212720	HSD11B2	dre-miR-146a
NM_212801	wu:fl33b06,zgc:77439	dre-miR-21
NM_212854	RND1	dre-miR-21
NM_212886	pgm2	dre-miR-34a
NM_212936	zgc:85889	dre-miR-34a
NM_213122	sox4a	dre-miR-34a
NM_213307	Atp5j	dre-miR-21
NM_213337	TMEM59L	dre-miR-21
NM_213396	fez1	dre-miR-34a
NM_213480	pank1a	dre-miR-21
NM_214687	gpm6ab	dre-miR-21
NM_214731	DACT1	dre-miR-462

Supplementary Table 8. Predicted interactions at 48hpf for downregulated miRNAs.

Target (RefSeq accession)	Gene Symbol	miRNAs (miRBase ID)
NM_001001849	Trpv6	dre-miR-190b
NM_001002122	ttc4	dre-miR-19a-3p
NM_001002199	zgc:91845	dre-miR-219-5p
NM_001002298	Asna1	dre-miR-7b
NM_001002309	mgc352611	dre-miR-219-5p
NM_001002455	Phlda3	dre-miR-19a-3p
NM_001002567	Mpv17l2	dre-miR-19a-3p
NM_001002687	zgc:91849	dre-miR-7b
NM_001003505	Stac3	dre-miR-7b
NM_001003509	GRWD1	dre-miR-7b
NM_001003583	OPTC	dre-miR-19a-3p
NM_001003608	nr2e1	dre-miR-219-5p
NM_001004637	zgc:101635	dre-miR-190b
NM_001006105	Golph3	dre-miR-19a-3p
NM_001007151	shbg	dre-miR-219-5p
NM_001008402	extl3	dre-miR-19a-3p,dre-miR-7b
NM_001008620	TSTA3,zgc:100864	dre-miR-19a-3p
NM_001013270	YES1	dre-miR-19a-3p
NM_001013273	CAPRIN2	dre-miR-19a-3p
NM_001013306	zgc:173721	dre-miR-19a-3p
NM_001013318	zgc:110779	dre-miR-219-5p
NM_001013521	Enah,zgc:103638	dre-miR-7b
NM_001013572	si:dkeyp-34c12.1	dre-miR-19a-3p
NM_001017665	zgc:112052	dre-miR-19a-3p,dre-miR-219-5p
NM_001017718	zgc:112178	dre-miR-219-5p
NM_001017809	zgc:110323	dre-miR-19a-3p
NM_001017853	zgc:110152	dre-miR-7b
NM_001020480	nmt1a	dre-miR-219-5p
NM_001020614	Eri1	dre-miR-19a-3p
NM_001020656	zgc:112165	dre-miR-190b
NM_001020714	zgc:113162	dre-miR-190b
NM_001025165	METAP1	dre-miR-190b
NM_001025181	zgc:114174	dre-miR-219-5p
NM_001025450	dnmt4	dre-miR-19a-3p,dre-miR-7b
NM_001025494	si:dkey-39a18.1	dre-miR-19a-3p
NM_001025509	si:dkey-159a18.3	dre-miR-7b
NM_001025550	si:ch211-261f7.2	dre-miR-19a-3p
NM_001029969	fynb	dre-miR-19a-3p
NM_001030000	dynll2a,dynll2b	dre-miR-19a-3p
NM_001030125	camk2g1	dre-miR-190b,dre-miR-219-5p
NM_001030285	EZR	dre-miR-219-5p
NM_001034019	kctd12.2	dre-miR-19a-3p
NM_001037413	zgc:123272	dre-miR-7b
NM_001037683	tgfbr1a	dre-miR-190b
NM_001038009	EPO	dre-miR-190b
NM_001039829	plagx	dre-miR-7b
NM_001039998	zgc:111880	dre-miR-7b
NM_001040369	zgc:136557	dre-miR-19a-3p
NM_001044884	si:ch211-222e23.7	dre-miR-19a-3p
NM_001044976	KIF14	dre-miR-19a-3p
NM_001045013	CHAF1A	dre-miR-7b
NM_001045064	hcfc1a	dre-miR-7b
NM_001045307	zgc:136367	dre-miR-19a-3p
NM_001045472	BBC3	dre-miR-219-5p
NM_001076564	zgc:154065	dre-miR-19a-3p
NM_001076569	zgc:153980	dre-miR-190b
NM_001076575	si:dkeyp-50f7.6,zgc:153725	dre-miR-19a-3p
NM_001076706	foxj1a	dre-miR-219-5p
NM_001076718	adam28	dre-miR-7b

Supplementary Table 8. continued...

Target (RefSeq accession)	Gene Symbol	miRNAs (miRBase ID)
NM_001076730	zgc:153610	dre-miR-19a-3p
NM_001076745	zgc:154071	dre-miR-190b,dre-miR-19a-3p
NM_001076748	rheb	dre-miR-219-5p
NM_001077259	zgc:154116	dre-miR-19a-3p
NM_001077387	Alkbh5	dre-miR-190b
NM_001079952	Tnip1	dre-miR-190b
NM_001079977	LOC796384,hmgcra	dre-miR-19a-3p
NM_001079981	ISOC1	dre-miR-219-5p
NM_001080029	arrdc3	dre-miR-19a-3p
NM_001080052	eya3	dre-miR-19a-3p
NM_001080202	si:ch211-103f16.2	dre-miR-19a-3p
NM_001080561	zgc:158645	dre-miR-7b
NM_001080599	zgc:158390	dre-miR-7b
NM_001080638	slc25a15a	dre-miR-190b
NM_001080662	zgc:158294	dre-miR-219-5p
NM_001080669	srpk1b	dre-miR-190b,dre-miR-219-5p
NM_001082832	si:dkey-7c18.24	dre-miR-7b
NM_001082837	Skp2	dre-miR-219-5p,dre-miR-7b
NM_001082921	zgc:162126	dre-miR-19a-3p
NM_001082998	zgc:158347	dre-miR-19a-3p,dre-miR-219-5p
NM_001089503	zgc:162977	dre-miR-190b,dre-miR-219-5p
NM_001089523	zgc:162267	dre-miR-19a-3p
NM_001089560	zgc:162208	dre-miR-19a-3p
NM_001099603	nkx2.9	dre-miR-19a-3p
NM_001100085	DPH5,LOC100003898	dre-miR-7b
NM_001102638	ms4a17a.4,ms4a17a.5	dre-miR-190b,dre-miR-219-5p
NM_001110393	Olig3,olig4	dre-miR-19a-3p
NM_001110458	Dis3l	dre-miR-19a-3p
NM_001111081	ghrb	dre-miR-7b
NM_001111171	zgc:171591	dre-miR-7b
NM_001111232	cbsA	dre-miR-19a-3p
NM_001113646	nkain4	dre-miR-19a-3p,dre-miR-7b
NM_001123312	MYST3	dre-miR-7b
NM_001128254	LOC567448	dre-miR-190b,dre-miR-19a-3p
NM_001145089	Camk2n1	dre-miR-19a-3p
NM_001145577	si:ch211-157h5.2	dre-miR-219-5p
NM_001145786	DNTTIP2	dre-miR-7b
NM_001161470	LOC325449,MT,Mt2	dre-miR-19a-3p
NM_001171587	prox3	dre-miR-19a-3p
NM_130944	dlc	dre-miR-19a-3p,dre-miR-219-5p,dre-miR-7b
NM_131025	CCND1	dre-miR-19a-3p,dre-miR-219-5p,dre-miR-7b
NM_131114	eve1	dre-miR-19a-3p
NM_131163	B2M	dre-miR-19a-3p
NM_131184	PAX5,pax2a	dre-miR-19a-3p
NM_131204	hsp47	dre-miR-219-5p
NM_131225	rx1	dre-miR-19a-3p,dre-miR-219-5p
NM_131266	cmyb	dre-miR-219-5p
NM_131275	rxrba	dre-miR-19a-3p
NM_131328	hsp90a.1	dre-miR-190b
NM_131510	CASP8	dre-miR-219-5p
NM_131600	hsf1	dre-miR-190b
NM_131613	Celf1	dre-miR-7b
NM_131671	atp1b1b	dre-miR-219-5p,dre-miR-7b
NM_131689	atp1a1a.3,atp1a1a.4	dre-miR-190b
NM_131809	cx44.1	dre-miR-19a-3p
NM_153673	UNC45B	dre-miR-19a-3p
NM_181437	PRL	dre-miR-219-5p
NM_182859	crabp2a	dre-miR-7b
NM_194363	mpp5a	dre-miR-7b

Supplementary Table 8. continued...

Target (RefSeq accession)	Gene Symbol	miRNAs (miRBase ID)
NM_194410	VAX1	dre-miR-19a-3p
NM_198067	Mmp2	dre-miR-19a-3p
NM_199215	CA2	dre-miR-190b,dre-miR-219-5p
NM_199547	ssb	dre-miR-19a-3p
NM_199611	arg2	dre-miR-19a-3p
NM_199832	tk1	dre-miR-190b,dre-miR-219-5p
NM_199950	socs3a	dre-miR-19a-3p,dre-miR-7b
NM_200051	c1galt1b	dre-miR-7b
NM_200085	SLC16A1	dre-miR-7b
NM_200099	dynll2a,dynll2b	dre-miR-190b,dre-miR-19a-3p,dre-miR-7b
NM_200107	zgc:64114	dre-miR-219-5p
NM_200321	RBM28	dre-miR-7b
NM_200340	PLEKHF1	dre-miR-19a-3p
NM_200425	lsm14a	dre-miR-190b,dre-miR-7b
NM_200432	zgc:63466	dre-miR-7b
NM_200473	UBE3C	dre-miR-19a-3p
NM_200487	tbc1d23	dre-miR-19a-3p
NM_200557	GOPC	dre-miR-19a-3p
NM_201006	ttc4	dre-miR-190b,dre-miR-19a-3p
NM_201091	lin28	dre-miR-19a-3p
NM_201175	oxsr1b	dre-miR-190b,dre-miR-19a-3p,dre-miR-219-5p,dre-miR-7b
NM_201185	bokb	dre-miR-19a-3p
NM_201214	eif3ja	dre-miR-219-5p
NM_201507	MAPK3	dre-miR-219-5p
NM_205593	zgc:158183	dre-miR-7b
NM_205668	zgc:77285	dre-miR-190b
NM_205747	irf11	dre-miR-7b
NM_212860	REN	dre-miR-190b
NM_212898	mettl11a	dre-miR-190b,dre-miR-219-5p
NM_212987	UBE2Q2	dre-miR-19a-3p
NM_213059	crsp7	dre-miR-19a-3p
NM_213099	WDR5	dre-miR-19a-3p
NM_213239	nr2f6b	dre-miR-219-5p
NM_213262	Eif5a2	dre-miR-190b,dre-miR-19a-3p
NM_213296	timp2b	dre-miR-7b
NM_213304	socs3b	dre-miR-19a-3p
NM_213353	SC4MOL	dre-miR-219-5p
NM_213385	LOC794952,VRK1	dre-miR-19a-3p
NM_213397	pfkfb3	dre-miR-190b,dre-miR-19a-3p
NM_213405	zgc:56630	dre-miR-19a-3p
NM_213419	MAP2K1	dre-miR-190b
NM_213499	Actr2	dre-miR-190b
NM_213519	SESN3	dre-miR-19a-3p

Supplementary Table 9. Function and Pubmed ID details about genes present in Figure 3.

miRNA	Target	Function	Pubmed ID
miR-34a	rhoad	heart contraction	21622831
miR-30a	trib3	heart looping	24292884
miR-30a	tmem2	cell migration involved in heart formation; embryonic heart tube morphogenesis; endocardial cushion development	21896630;21896629
miR-30a	sox7	artery development; blood vessel development	23818617;25834021
miR-30a	robo1	endocardial progenitor cell migration to the midline involved in heart field formation	21385766
miR-30a	nrp2b	vasculogenesis	21385766
miR-30a	mbnl2	heart development	21385766
miR-30a	dlc	dorsal aorta development	21385766
miR-30a	cbfb	heart contraction	21385766
miR-30a	cav1	cardiac muscle cell proliferation	21385766
miR-30a	camk2d1	heart jogging	21385766
miR-30a	bmpr2a	heart jogging; heart looping	21385766
miR-10d	bmpr2a	heart jogging; heart looping	21385766
miR-10d	mef2ca	cardiac muscle cell development; cardiac muscle cell differentiation; heart development	21385766
miR-10d	rbpja	artery development	21385766

Supplementary Table 10. Function and Pubmed ID details about genes present in Figure 4.

miRNA	Target	Function	Pubmed ID
miR-146a	lpar1	angiogenesis	21971049
miR-146a	hoxb5b	heart development	23990796
miR-462	gsk3b	angiogenesis; heart jogging; heart looping	25056693;17683539
miR-7b	dlc	dorsal aorta development	24598161
miR-7b	mpp5a	embryonic heart tube development	16319113
miR-19a	ttc4	determination of heart left/right asymmetry	25860617
miR-19a	prox1b	lymphangiogenesis	20976189
miR-19a	dlc	dorsal aorta development	24598161
miR-19a	unc45b	cardiac muscle tissue development	17189627
miR-21	ndrg4	cardiac muscle cell proliferation; embryonic heart tube development; heart looping	18407257
miR-21	igfbp2a	angiogenesis; heart development	15618288
miR-190b	camk2g1	heart jogging	20630945
miR-190b	ttc4	determination of heart left/right asymmetry	25860617
